# New zeolite-like RUB-5 and its related hydrous layer silicate RUB-6 structurally characterized by electron microscopy

**DOI:** 10.1107/S2052252520003991

**Published:** 2020-04-21

**Authors:** Yaşar Krysiak, Bernd Marler, Bastian Barton, Sergi Plana-Ruiz, Hermann Gies, Reinhard B. Neder, Ute Kolb

**Affiliations:** a Institute of Inorganic Chemistry and Analytical Chemistry, Johannes Gutenberg University, Duesbergweg 10-14, Mainz D-55128, Germany; b Department of Materials and Geoscience, Technische Universität Darmstadt, Petersenstrasse 23, Darmstadt D-64287, Germany; c Department of Structure Analysis of the Institute of Physics, Czech Academy of Sciences, Cukrovarnická 10/112, Prague 162 00, Czech Republic; d Departure of Geology, Mineralogy and Geophysics, Ruhr University Bochum, Universitätsstrasse 150, Bochum D-44801, Germany; e LENS, MIND/IN2UB, Engineer department: Electronics section, Universitat de Barcelona, Martí i Franquès 1, Barcelona 08028, Spain; f Chair for Crystallography and Structural Physics, Friedrich-Alexander-Universität Erlangen-Nürnberg, Staudtstrasse 3, Erlangen D-91058, Germany

**Keywords:** 3D electron diffraction, exit wave reconstruction, diffuse scattering, stacking faults, electron crystallography, framework-structured solids, microporous materials, polymorph prediction, computational modelling, inorganic materials

## Abstract

This study made use of a recently developed combination of advanced methods to reveal the atomic structure of a disordered nanocrystalline zeolite using exit wave reconstruction, automated diffraction tomography, disorder modelling and diffraction pattern simulation. By applying these methods, it was possible to determine the so far unknown structures of the hydrous layer silicate RUB-6 and the related zeolite-like material RUB-5. The structures of RUB-5 and RUB-6 contain the same dense layer-like building units (LLBUs). In the case of RUB-5, these building units are interconnected via additional SiO_4/2_ tetrahedra, giving rise to a framework structure with a 2D pore system consisting of intersecting 8-ring channels. In contrast, RUB-6 contains these LLBUs as separate silicate layers terminated by silanol/sil­oxy groups. Both RUB-6 and RUB-5 show stacking disorder with intergrowths of different polymorphs. The unique structure of RUB-6, together with the possibility for an interlayer expansion reaction to form RUB-5, make it a promising candidate for interlayer expansion with various metal sources to include catalytically active reaction centres.

## Abstract 2

RUB-5 and its related hydrous layer silicate RUB-6 were synthesized in the 1990s, but so far their structures have remained unknown due to their low crystallinity and disorder. The combination of 3D electron diffraction, X-ray powder diffraction, high-resolution transmission electron microscopy, structural modelling and diffraction simulations has enabled a comprehensive description of these two nanomaterials, revealng a new framework topology and a unique silica polymorph.

## 1. Introduction   

Zeolites are tetrahedrally connected aluminosilicate frameworks with nanometre-sized pores and channels. They are important due to their widespread applications in catalysis, adsorption and ion exchange (Millini *et al.*, 2017 ▸). Frameworks are constructed from [TO_4_] tetrahedra, which are interconnected via oxygen atoms to form a 3D network (Li & Yu, 2014 ▸). Zeolites can be synthesized under hydro­thermal conditions from mixtures containing a silica source, a base, a cation source, water and, in many cases, an additional element occupying the T positions in the framework. Various synthesis approaches have been developed to prepare zeolites with new topologies, large pores and functional groups. In recent work, new zeolites or microporous silicates could be synthesized by topotactic condensation and interlayer expansion reactions of hydrous layer silicates (HLSs) (Marler & Gies, 2012 ▸; Gies *et al.*, 2016 ▸). Different metal cations can be introduced by interlayer expansion of HLSs connecting the layers via Me—O bonds (Wang *et al.*, 2009 ▸; Gies *et al.*, 2011 ▸; Bian *et al.*, 2015 ▸). These materials have a high potential for applications in catalysis.

Knowledge of the atomic structure is essential for tuning the properties of such microporous materials, but often synthetic zeolites form polycrystalline powders, with crystal sizes below 10 µm, which are not appropriate for single-crystal X-ray diffraction experiments. In many cases, X-ray powder diffraction (XRPD) has been successfully used for structure determination and refinement. Furthermore, solid-state nuclear magnetic resonance (SSNMR) spectroscopy, which probes short-range order, can yield complementary information to diffraction experiments which probe long-range order and periodicity (Brouwer, 2008 ▸). ^29^Si MAS NMR spectra allow us to determine the connectivity of the [SiO_4_] tetrahedra and calculate the *Q* ^3^:*Q* ^4^ ratio of the silicon atoms. ^13^C MAS NMR and ^1^H MAS NMR spectroscopy yield information on organic species within the zeolite framework or inside the interlayer spaces of the HLSs, and on protons involved in hydrogen bonding to silanol/sil­oxy groups.

The structures of HLSs are generally characterized by weak interactions between silicate layers, leading to a slight or even pronounced stacking disorder of the layers. Moreover, the condensation of HLS into zeolites often results in nanometre- and submicrometre-sized crystals with poor crystallinity. Thus, peak broadening and poorly resolved XRPD patterns make structure determination difficult or even impossible. Consequently, structure determination often demands more than the combination of XRPD and SSNMR. Over the last decade, 3D electron diffraction (3D ED) has been established as an alternative method for *ab initio* structure determination of nanometre-sized crystals (Gemmi *et al.*, 2019 ▸), including complex zeolites and layered silicates (Mugnaioli & Kolb, 2015 ▸; Yun *et al.*, 2015 ▸). Based on the development of automated electron diffraction tomography (ADT) (Kolb *et al.*, 2007 ▸), it is now possible to sample a substantial fraction of the 3D reciprocal lattice of nanocrystals in a relatively short period of time of less than 1 h. At the same time, the applied electron dose is significantly reduced in comparison with classical zone axis electron diffraction. Using ADT, a non-oriented single nanocrystal is tilted by a constant increment (usually 1°) around the goniometer axis and a 2D diffraction pattern is recorded at each tilt angle. The rotation electron diffraction (RED) (Zhang *et al.*, 2010 ▸) method follows a similar approach, using a combination of electron beam and goniometer tilt. A further reduction of electron beam damage is still desirable, especially for the characterization of proteins and other beam-sensitive materials. The advent of CMOS cameras that do not require the conversion of electrons to photons (so-called direct detection) enabled Nederlof *et al.* and Gemmi *et al.* to introduce an advanced recording method for 3D ED. The recording times are reduced to minutes by tilting the nanocrystal continuously during pattern acquisition with no need for sequential crystal particle tracking (Nederlof *et al.*, 2013 ▸; Nannenga *et al.*, 2014 ▸; Gemmi *et al.*, 2015 ▸). Recently, the capabilities of this novel technique have been demonstrated on the new zeolite ITQ-58 (Simancas *et al.*, 2016 ▸). Furthermore, for a precise recording of streaks and broadened reflections due to structural disorder, the diffraction space can be fine-scanned using direct detection cameras (Nederlof *et al.*, 2013 ▸; Gemmi *et al.*, 2015 ▸; Plana-Ruiz *et al.*, 2020 ▸).

Regardless of the acquisition method used, dedicated programs such as *eADT* (former *ADT3D*), *PETS*, *RED* and *EDT-PROCESS* (Kolb *et al.*, 2019 ▸; Palatinus, 2011 ▸; Wan *et al.*, 2013 ▸; Oleynikov, 2011 ▸; Gemmi & Oleynikov, 2013 ▸) are employed to reconstruct the 3D reciprocal lattice, which allows the determination of cell parameters, the space group and furthermore the extraction of intensities suitable for structure solution by direct methods, charge flipping or simulated annealing in analogy to X-ray diffraction experiments. The ability to also reconstruct the raw diffraction pattern into 3D data simplifies the interpretation of disorder, phase mixtures and twinning (Kolb *et al.*, 2011 ▸, 2019 ▸; Su *et al.*, 2014 ▸; Cichocka *et al.*, 2018 ▸; Mayorga-Martinez *et al.*, 2018 ▸).

Disorder is a common feature in zeolite structures, and different stacking sequences of the same layer can result in distinct polymorphs. Zeolite beta (Newsam *et al.*, 1988 ▸; Higgins *et al.*, 1988 ▸), SSZ-31 (van Koningsveld & Lobo, 2003 ▸), SSZ-57 (Baerlocher *et al.*, 2011 ▸), ZSM-48 (Lobo & Koningsveld, 2002 ▸) and ITQ-39 (Willhammar *et al.*, 2012 ▸) are examples of intergrowth families, with zeolite beta being the most important representative. High-resolution transmission electron microscopy (HRTEM) comprises a powerful technique for studying such disordered nanocrystals at the atomic scale because, in contrast to diffraction techniques, it does not require translational symmetry. Through HRTEM imaging, stacking faults and defects can be visualized directly in real space (Kisielowski *et al.*, 2008 ▸).

However, since HRTEM yields only 2D information and requires electron doses of hundreds to thousands of electrons per Å^2^ (Barton *et al.*, 2012 ▸) which are often not tolerated by beam-sensitive zeolites, a combination of HRTEM with different methods is required for *ab initio* description of 3D atomic structures. In the case of ITQ-39, Wilhammer *et al.* were able to develop a procedure for solving 3D structures of intergrown nanocrystals by a combination of electron diffraction with HRTEM through-focal series, RED and crystallographic image processing (Willhammar *et al.*, 2012 ▸; Kapaca *et al.*, 2017 ▸).

Recently, we presented an alternative approach in which we first determine the average crystal structure of a material based on ADT data using direct methods for *ab initio* structure analysis and subsequently compare the resulting model with structural images produced by exit wave reconstruction (Krysiak *et al.*, 2018 ▸). Possible deviations from the periodic structure, such as layer shifts, can be determined and then modelled, for example, with the *DISCUS* software package (Proffen & Neder, 1997 ▸). These simulated electron diffraction patterns can in turn be compared qualitatively with the reconstructed experimental reciprocal space. Using this method, we were able to demonstrate the possibility of disorder analysis on nanoparticles in a quantitative way (Krysiak *et al.*, 2018 ▸).

In this article, we report on the successful structure determination of two disordered materials possessing a new type of silica layer, which is present in the hydrous layer silicate RUB-6 and its corresponding framework silicate RUB-5. The structure of RUB-5 including disorder and intergrowth was solved and analysed in detail by applying the combination of 3D ED, XRPD, HRTEM, structural modelling and diffraction pattern simulations.

## 2. Experimental   

### 2.1. Synthesis   

The two RUB-5 and RUB-6 samples used in this study were synthesized at 160°C from reaction mixtures of 0.8SiO_2_/0.2LiOH/0.2B(OH)_3_/1OA/55.5H_2_O using 2-butyl-2-ethyl-1,5pentandi­amine (RUB-5, 98 days) and 4-amino­methyl-piperidine (RUB-6, 49 days) as organic additives (OAs). The synthesis was carried out as described by Marler *et al.* (2020 ▸).

### 2.2. Electron diffraction   

#### 2.2.1. Automated electron diffraction tomography   

The powdered samples were dispersed in ethanol using an ultrasonic bath and sprayed on a carbon-coated copper grid using an ultrasound sonifier (Mugnaioli *et al.*, 2009 ▸) for transmission electron microscopy (TEM), electron dispersive X-ray spectroscopy (EDXS) and automated electron diffraction tomography (ADT) investigations. TEM, EDX and ADT measurements were carried out with an FEI TECNAI F30 S-TWIN transmission electron microscope equipped with a field emission gun and working at 300 kV. TEM images and nano electron diffraction (NED) patterns were taken with a CCD camera (16-bit 4096 × 4096 pixel GATAN ULTRASCAN4000) and acquired by *Gatan Digital Micrograph* software. Scanning transmission electron microscopy (STEM) images were collected by a FISCHIONE high-angular annular dark field (HAADF) detector and acquired by *Emispec ES Vision* software. 3D ED data were collected using an automated acquisition module developed for FEI microscopes (Kolb *et al.*, 2007 ▸). For high-tilt experiments all acquisitions were performed with a FISCHIONE tomography holder. A condenser aperture of 10 µm and mild illumination settings (gun lens 8, spot size 6) were used in order to produce a semi-parallel beam of 100 nm diameter (4.15 e^−^ Å^−2^s^−1^). Crystal position tracking was performed in microprobe STEM mode and NED patterns were acquired sequentially in steps of 1°. Tilt series were collected within a total tilt range of up to 120°. ADT data were collected with electron beam precession (precession electron diffraction, PED) (Vincent & Midgley, 1994 ▸). PED was used to improve reflection intensity integration quality (Mugnaioli *et al.*, 2009 ▸), and was performed using a Digistar unit developed by NanoMEGAS SPRL. The precession angle was kept at 1.0°. The *eADT* software package was used for 3D ED data processing (Kolb *et al.*, 2011 ▸). *Ab initio* structure solution was performed assuming the kinematic approximation *I* ≃ |*F*_*hkl*_|^2^ by direct methods implemented in the program *SIR2014* (Burla *et al.*, 2015 ▸). The calculation of difference Fourier maps was performed using the software *SIR2014* (Burla *et al.*, 2015 ▸). Scattering factors for electrons were taken from the work by Doyle & Turner (1968 ▸).

#### 2.2.2. Fast-ADT   

Fast-ADT acquisition was performed on the FEI Tecnai F30 S-Twin TEM. Matlab-based *Fast-ADT* software using the Stingray F-145B CCD optical camera (1388 × 1038 pixels) provided by Allied Vision GmbH was used to record the continuous 3D ED from the small fluorescent screen. First, a pre-tilt scan of the goniometer stage was applied in 5° steps between −50 and 60° and images were taken at every discrete tilt step. The non-linear movement of the crystal with respect to the tilt angle was estimated by cross-correlation calculation. A 50 µm condenser aperture (spot size 7 and gun lens 4) was set to produce a 400 nm beam. A NanoMEGAS P1000 unit was used to generate the beam shift required to follow the crystal while the stage is continuously tilted. A continuous dataset was acquired between −50 and 60° with a tilt velocity of 1.75° s^−1^ controlled by the FEI CompuStage dialogue and the camera exposure time was set to 30.4 ms, although the camera electronics limit the frame rate to a maximum of 16.07 frames per second (fps). A total of 1018 diffraction patterns were continuously acquired during the time period of approximately 1 min, giving an integrated angular range for each diffraction pattern of 0.053°. The obtained diffraction patterns were geometrically corrected through projective transformations available in the Matlab-based *Fast-ADT* software. One diffraction pattern was taken every 0.2° to reduce the calculation time for data processing without significantly decreasing the data quality compared with the use of all frames. A final image stack of 820 × 820 × 55 pixels was used for the reconstruction of the observable diffraction space using the *eADT* software. Matlab-based and self-developed scripts were used to extract the desired zone axes and intensity profiles. Fast-ADT acquisition is reported in more detail by Plana-Ruiz *et al.* (2020 ▸).

### 2.3. HRTEM   

TEM exit wave reconstruction was realized by focal series recorded using the FEI F30 ST described above, without an aberration corrector, under suitable high-resolution TEM conditions. In total, 20 images were recorded at a primary magnification of 790 000 with 6 nm focal increment, thus covering a focal range of 114 nm including Gaussian focus. The accumulated dose per focal series was 1200 e^−^ Å^−2^. The images were hardware-binned by 2, followed by an additional software binning by 2 resulting in 1*k* × 1*k* images with a physical pixel size of 0.576 Å. After image alignment, a 350^2^ pixel area was chosen for exit wave reconstruction, employing a Gerchberg–Saxton algorithm written and implemented in Python. Residual axial aberrations were corrected by an automated downhill simplex minimization routine also implemented in Python (Lehmann, 2000 ▸). Simulated TEM exit waves were generated using a multislice algorithm included in the *Dr. Probe* software package (Barthel, 2018 ▸).

### 2.4. Structure refinement of the average structures of RUB-5 and RUB-6   

Powder XRD data were recorded with a Siemens D5000 powder diffractometer in modified Debye–Scherrer geometry using Cu *K*α_1_ radiation (λ = 1.54059 Å). Samples were sealed in borosilicate glass capillaries with a diameter of 0.3 mm to avoid preferred orientation of the plate-like crystals and the loss or uptake of water by the samples. The diffractometer was equipped with a curved germanium (111) primary monochromator and a Braun linear position-sensitive detector covering a 2θ range of 6°.

The average structures of RUB-5 and RUB-6 (based on the structure models as determined by the ADT method) were refined from the powder XRD data using the *FullProf 2K* program (Rodríguez-Carvajal, 2006 ▸). No absorption correction was necessary. The refinements of both models were performed in the space group *C*2. Soft distance restraints were used for *d*(Si—O) = 1.61 (1), *d*(Si⋯Si) = 3.10 (4), *d*(O⋯O) = 2.615 (3), *d*(C—C) = 1.54 (1), *d*(C—N) = 1.47 (1) and *d*(C⋯N, next-next neighbour) = 2.45 (2) Å. Displacement parameters (*B*_iso_) were fixed at chemically meaningful values: 1.0 Å^2^ for Si, 2.0 Å^2^ for O and 4.0 Å^2^ for C and N. Anisotropic Lorentzian size broadening – modelled using spherical harmonics – was assumed to apply to the peak shapes of RUB-5 and RUB-6. Size model 15 (Laue class: 2/*m*) was used comprising nine parameters. The details of the data collection and the results of the structure refinements are summarized in Table S1 of the supporting information. Careful analyses of the powder patterns revealed that, in the case of the RUB-5 sample, a few very weak reflections from the MFI-type impurity were also present (between 7 and 10° 2θ). In the case of RUB-6 a small amount of MTW-type zeolite was present. Therefore the structure refinements included either the known structure of ZSM-5 (Kokotailo *et al.*, 1978 ▸) as a second phase (refinement of RUB-5) or the known structure of ZSM-12 (LaPierre *et al.*, 1985 ▸) (refinement of RUB-6). In both cases, only the lattice parameters and the scale factors of the impurity phases were refined (see Table S1).

### 2.5. Disorder modelling and diffraction simulations   

After determining the average structure of RUB-5, the disordered real structure was studied: in order to simulate the diffuse scattering from RUB-5, complex stacking disordered structures based on three different layers were modelled and the corresponding electron diffraction patterns were calculated with the *DISCUS* software package (Proffen & Neder, 1997 ▸). A slice of the ordered (average) structure of RUB-5 extending along the *a* and *b* directions was used as layer α. The β layer was created by applying two symmetry operations on the α layer: first, a rotation of 180° around the *a* direction applied at the origin [*x*, 1/2, 1/2]; second, a shift of Δ*y* = −0.293 along *b*. Layer γ was built by deleting a thin layer δ of silicon atoms from the RUB-5 structure [marked areas in Figs. 2(*b*) and 3(*a*)]. Stacking sequences of the layers α, β and γ were built with the *stack module* of *DISCUS*. For the calculation of the 2D electron diffraction (ED) pattern, the individual layers were simulated as a 10 × 10 × 1 supercell; 40 of these layers stack along the *c* direction. Atom positions of layered structures can be created through a convolution product of the atom positions within a single-layer type with the list of the layer positions. The diffraction pattern in turn is the regular product of the Fourier transform of the atom positions of a single layer with the Fourier transform of the list of layer origins. These individual Fourier transformations take much less time to calculate than the Fourier transform of the full crystal model (Zhao *et al.*, 2017 ▸). For the calculation of 1D line profiles, 40 individual layers (1 × 1 × 1 supercells) were stacked along the *c* direction. The corresponding line profiles were calculated in the interval −9 ≤ *l* ≤ +9 for each value of *h* and *k*. The stackings and simulations were repeated ten times and merged for each individual ED pattern and 1D line profile to improve the statistics.

### 2.6. Tiling and visualization of crystal structures   

The tiling data were calculated with *TOPOS* (4.0, Blatov, 2012 ▸) while structure plots were created with *VESTA* (Momma & Izumi, 2011 ▸).

## 3. Results and discussion   

### 3.1. Synthesis and characterization   

The synthetic work, spectroscopic analysis and chemical classification are mainly discussed in another report (Marler *et al.*, 2020 ▸). The majority of the particles from RUB-5 and RUB-6 samples observed by scanning electron microscopy (SEM) consist of thin plate-like crystals that are highly agglomerated. The crystal size is approximately isometric in two directions (ranging from 2 to 10 µm), and the plate thickness is around 0.1 µm. Thereby, the crystals are too small for single-crystal X-ray diffraction analysis. The XRPD pattern showed that both compounds are crystalline in principal, but the structure solution or even lattice indexing have been impossible for the last 25 years due to the strong peak overlapping. The strong broadening of the diffraction intensities hints to additional disorder and smaller crystal sizes than observed through SEM. It should be mentioned here that the crystallinity of RUB-6 is lower than for RUB-5 and the ^29^Si MAS NMR spectra of RUB-6 show additional *Q* ^3^ peaks in contrast to RUB-5. This is an indication that RUB-5 is a framework silicate, whereas RUB-6 contains silanol/sil­oxy groups which are part of the silicate layers.

### 3.2. Structure determination of RUB-5   

#### 3.2.1. 3D electron diffraction   

We first investigated the structure of RUB-5 through EDX, ADT and HRTEM with an FEI F30 microscope operating at 300 kV. The single crystals observed by microprobe STEM imaging (see Fig. S1 of the supporting information) have face sizes of about 1 µm^2^ down to 0.01 µm^2^. The plate-like morphology of the crystals induced a strong preferred orientation of the particles on the grid. This makes upstanding crystals extremely rare. One example is given in Fig. S1, which shows a plate thickness around 15–20 nm. The composition measured by EDX, *n*(Si):*n*(O) ≃ 1/2, is the same for all of the collected EDX spectra; an example is shown in Fig. S3.

**Figure 1:**
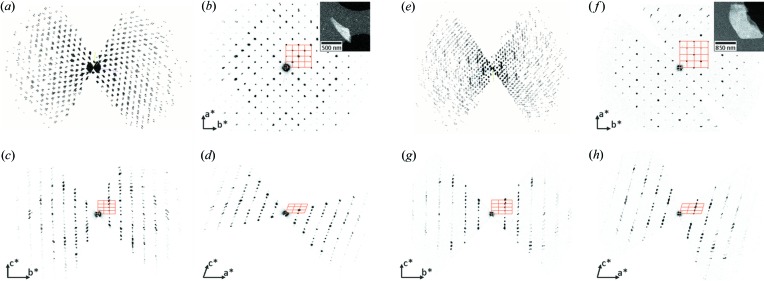
Reconstructed 3D diffraction data obtained by ADT. RUB-5 (left): (*a*) view along the goniometer axis, (*b*) *hk*0 section, (*c*) 0*kl* section and (*d*) *h*0*l* section. RUB-6 (right): (*e*) view along the goniometer axis, (*f*) *hk*0 section, (*g*) 0*kl* section and (*h*) *h*0*l* section.

A couple of ADT datasets were collected from isolated particles and reconstructed in 3D diffraction data. For each measured particle, the diffraction data showed the same lattice with diffuse scattering along the shortest reciprocal direction. In addition, each of the measured particles is composed of several crystallites. These circumstances made the determination of the crystal lattice and symmetry difficult. For instance, the diffraction volume shown in Fig. 1 ▸ delivered a primitive lattice with the parameters *a* = 7.83, *b* = 7.72, *c* = 19.03 Å, α = 100.7, β = 103.2 and γ = 91.1°. The lattice is very close to a tetragonal body-centred setting (*a* = 7.72, *b* = 7.72, *c* = 36.37 Å, α = 91.6, β = 89.2, γ = 91.1°), but analysis of the internal residual after merging symmetry-equivalent reflections (*R*_sym_ = 0.294) indicates a lower symmetry.

**Figure 2:**
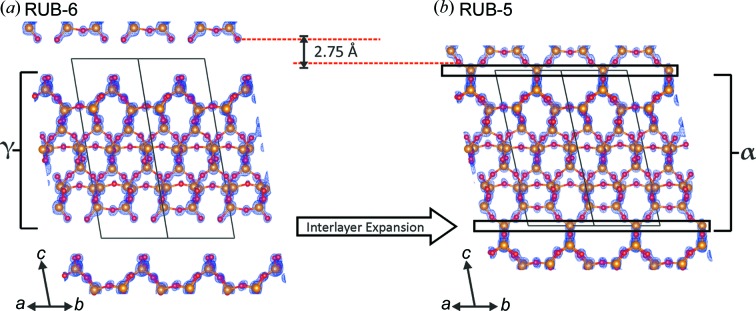
Potential map, calculated with *JANA2006* (Petříček *et al.*, 2014 ▸), of the structure solution combined with the structure model projected along [−1−10] for (*a*) RUB-6 and (*b*) RUB-5. Layer γ is marked in (*a*) and layer α is marked in (*b*). Silicon atoms are shown in orange and oxygen atoms in red.

Eventually, the lattice was found to be base-centred monoclinic with the parameters *a* = 10.88, *b* = 11.10, *c* = 18.92 Å, α = 88.6, β = 106.1 and γ = 89.2°, confirmed by a lower residual for merged symmetry-equivalent reflections (*R*_sym_ = 0.167, Laue class 2/*m*). The extinction group was determined as *C*1–1, consistent with space groups *C*2, *Cm* and *C*2/*m*. For structure solution, lattice parameters of a LeBail fit of XRPD data given in Table 1 ▸ were used. *Ab initio* structure solution, as shown in Fig. 2 ▸, converged to a final residual *R*_F_ of 0.171. The electron density map has 12 strong maxima (from 3.61 to 2.33 e^−^ Å^−3^) corresponding to 12 independent silicon atoms. Taking into account that RUB-5 is a framework silicate, 19 maxima (from 2.15 to 1.22 e^−^ Å^−3^) and 1 weaker maximum (0.57 e^−^ Å^−3^) could be assigned to 20 oxygen atoms. Eight of the weakest 9 maxima (from 1.21 to 0.56 e^−^ Å^−3^) and 1 higher maximum (1.29 e^−^ Å^−3^) were not taken into account. One missing oxygen was added manually.

#### 3.2.2. Rietveld refinement of the average structure   

For a further refinement of the structure solution based on ADT data, a Rietveld refinement was carried out. The XRD powder pattern exhibits significant anisotropic broadening of the Bragg reflections indicating a disordered structure. It is instructive to compare the refined values of the full width at half-maximum (FWHM) for reflections at similar diffraction angles (reflections around 2θ = 25 ± 2°). The sharpness of the *hk*0 reflections (FWHM_220_ = 0.12°, FWHM_130_ = 0.13°) indicates that the structure is well ordered within the layer-like building unit (LLBU) (*ab* plane). All Bragg reflections with indices *h* ≠ 0 and/or *k* ≠ 0 and |*l*| > 1 are particularly broad: FWHM_023_ = 0.32°, FWHM_202_ = 0.32°, FWHM_-204_ = 0.30°, FWHM_114_ = 0.28°, FWHM_-115_ = 0.29°, FWHM_-223_ = 0.28°, FWHM_024_ = 0.33°. The fact that, for example, the 023 and 202 reflections possess a larger FWHM than the 005 reflection (FWHM = 0.24°) indicates that the plate-like morphology of the crystals (thickness of the crystals *ca* 0.1 µm along the *c* axis) is not the only reason for the observed anisotropic line broadening.

For the structure refinement, the atomic coordinates as determined by the ADT method were used as a starting model. The Rietveld refinement of the structure in space group *C*2 converged to residual values *R*_Bragg_ = 0.033 and *R*_F_ = 0.031 confirming the structure model (see Fig. S5). It was, however, not possible to account completely for the anisotropic broadening of the peaks; therefore, the profile fit is less good (χ^2^ = 3.3). Atomic coordinates, displacement parameters and occupancy factors are listed in Table S2.

The structure refinement includes three additional oxygen atoms (with occupancy factors of 0.6, 1 and 0.9) representing three extra-framework electron density maxima (E1, E2, E3) which were detected by difference Fourier analysis. Two of these maxima, E1 and E2, are located in the channel-like voids while the third maximum, E3, is located amid the atoms of the dense α layer with unrealistic E3—O (framework) distances of only 1.9 Å. Since the thermal analysis, the electron microprobe analysis and the FTIR spectrum have given no indication of water molecules in the structure (Marler *et al.*, 2020 ▸), it is assumed that these electron density maxima are generated by the disordered real structure of RUB-5, which is discussed later in this article.

#### 3.2.3. Description of the structure   

RUB-5 has a framework density of 22.0 silicon atoms per 1000 Å^3^ and represents a pure silica ‘zeolite’ of very high density.

**Figure 3:**
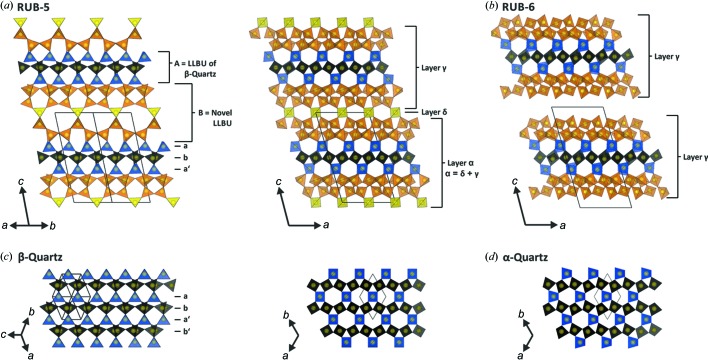
Visualization of crystal structures of (*a*) RUB-5 viewed along [−1−10], [0−10], (*b*) RUB-6 (illustrated without organic molecules) viewed along [0−10], (*c*) β-quartz viewed along [−1−1−1] and [00−1], and (*d*) α-quartz viewed along [−1−1−1]. LLBUs (labels α, β, γ) and subunits (labels *a*, *b*, *a*′, *b*′) are marked. The relations between layer γ and layer α are illustrated in (*a*) and (*b*).

The structure can be depicted in two ways: (i) Consisting of an alternation of the LLBU γ [see Figs. 2 ▸(*a*) and 3 ▸(*a*)] and a layer of additional interconnecting SiO_4/2_ tetrahedra [layer δ, marked yellow in Fig. 3 ▸(*a*)]; or (ii) an alternation of two other subunits. One subunit is topologically similar to the structure of quartz (LLBU *A*), whereas the other layer-like subunit (LLBU *B*) has no analogies to other notable LLBUs [Fig. 3 ▸(*a*)]. While the dense LLBU *A* explains the high density of RUB-5 {the structure of β-quartz viewed along [−1−1−1] is illustrated in Fig. 3 ▸(*c*)}, LLBU *B* represents the porous part of RUB-5. LLBU *B* forms a 2D pore system of intersecting 8-ring channels extending perpendicular to the *c* axis. The effective diameters are 3.8 × 4.2 Å for both channels.

**Figure 4:**
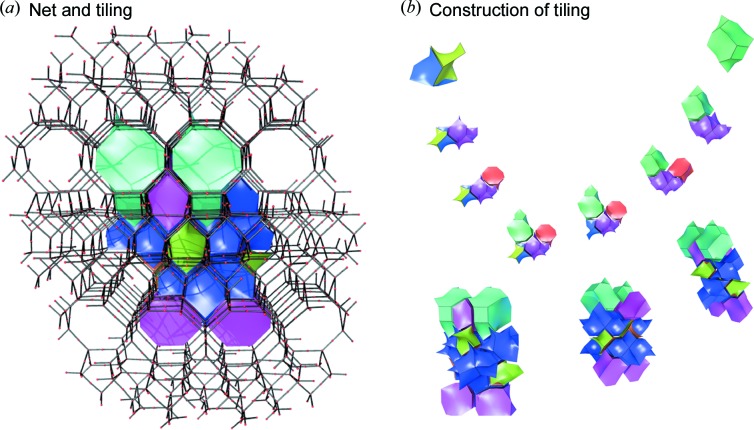
Details of the net and tiling construction of RUB-5. (*a*) Tiling with edges and vertices of the net. (*b*) Representation of the topology through the successive composition of the individual tiles, slightly shrunk for clarity, to the entire structure of RUB-5.

A tile representation of the RUB-5 framework is given in Fig. 4 ▸ and illustrates that RUB-5 has quite a complex structure with partly very irregular tiles. It should be noted that the topology of this silica framework is unique and has not been observed in any other zeolites, and thus represents a new silica polymorph (Baerlocher & McCusker, 2017 ▸).

### 3.3. Structure determination of RUB-6   

In a next step, we investigated the structure of RUB-6 through EDX and ADT. The particle morphology observed by microprobe STEM imaging is similar to RUB-5, showing plate-like morphology with face sizes around 1 µm^2^ down to 0.01 µm^2^ (see Fig. S2). Therefore the particles also have a preferred orientation on the grid. The chemical composition measured by EDX, *n*(Si):*n*(O) ≃ 1/2, is the same for all of the collected EDX spectra. An example is shown in Fig. S4.

#### 3.3.1. 3D electron diffraction   

RUB-6 is clearly more beam sensitive than RUB-5. To counteract the short lifetime of RUB-6 the electron beam dose was reduced to a value of 1.26 e^−^ Å^−2^s^−1^. ADT experiments coupled with precession electron diffraction (PED) were performed on isolated particles and the 3D diffraction volumes were reconstructed. The reflections could be indexed with a monoclinic base-centred lattice with *a* = 10.73, *b* = 11.22, *c* = 21.38 Å α = 90.5, β = 103.4 and γ = 89.4°. A LeBail refinement on XRPD data confirms, under consideration of the scale factor based on the effective camera length, the lattice determined by ADT. Details are shown in Table 1 ▸. Similar to RUB-5, the extinction group was determined as *C*1–1, consistent with space groups *C*2, *Cm* and *C*2/*m*. The structure was solved in space group *C*2 (*R*_sym_ = 0.132, Laue class 2/*m*) based on the assumption that the structure of RUB-6 is very closely related to that of RUB-5.

The *ab initio* structure solution, as shown in Fig. 2 ▸(*a*), has a final residual *R*_F_ of 0.141. The electron density map has 9 strong­est maxima (from 2.30 to 1.35 e^−^ Å^−3^) and 1 weaker maximum (1.19 e^−^ Å^−3^) corresponding to 10 independent silicon atoms. A total of 20 of the next 21 maxima (from 1.34 to 0.67 e^−^ Å^−3^) and 2 weaker maxima (0.62 and 0.49 e^−^ Å^−3^) corresponded to 22 oxygen atoms. Out of the weakest 9 maxima (from 0.65 to 0.36 e^−^ Å^−3^), 7 were not taken into account.

#### 3.3.2. Rietveld refinement of the average structure   

As in the case of RUB-5, for the refinement of the RUB-6 structure, the atomic coordinates of the silicon and oxygen atoms as determined by the ADT method were used as a starting model. In a later stage of the refinement, the positions of the carbon and nitro­gen atoms of the organic molecule were determined from difference Fourier maps. Due to the limited crystallinity (structural order) of the material, the displacement parameters were fixed at chemically meaningful values.

**Figure 5:**
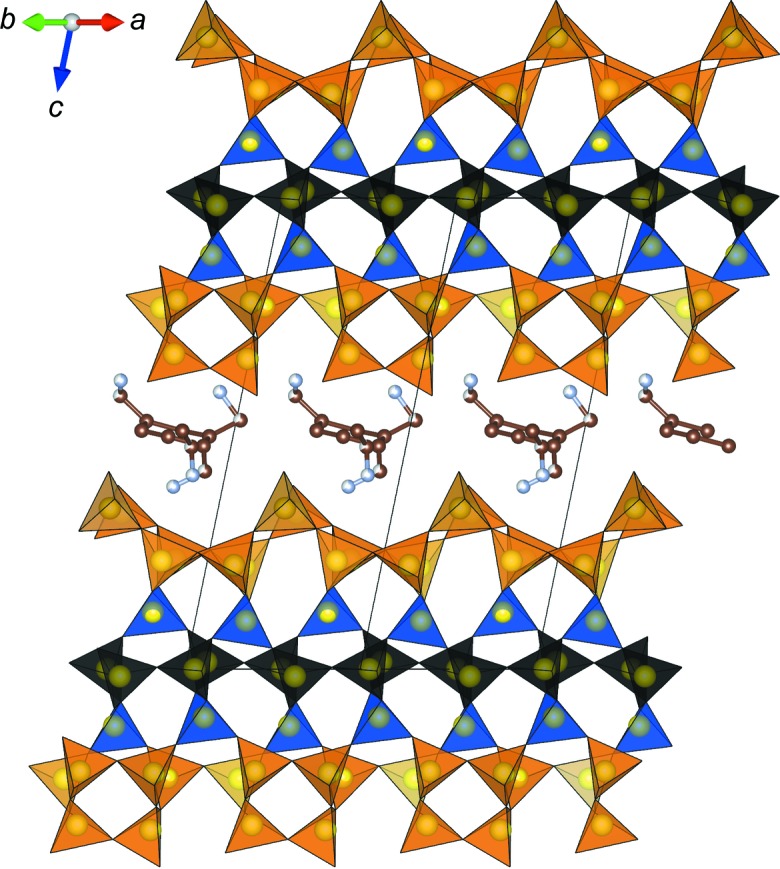
Projection of the (average) structure of RUB-6 along [110] (yellow = Si, brown = C, grey = N). Oxygen atoms have been omitted for clarity.

The Rietveld refinement of the structure in space group *C*2 converged to residual values *R*_Bragg_ = 0.026 and *R*_F_ = 0.025, (χ^2^ = 3.7) (see Fig. S6). The broad reflections in the powder diffractogram of RUB-6 indicate the presence of considerable disorder which is not accounted for by the refined average structure of RUB-6. Nevertheless, the resulting bond lengths and angles confirm the general features of the structure model. Moreover, in agreement with the results of the thermal analysis, FTIR spectroscopy and ^13^C NMR spectroscopy, the refinement led to the approximate location of the disordered organic cation/molecule and confirmed that, in the case of RUB-6, the γ layers are intercalated by organic compounds (Marler *et al.*, 2020 ▸). Fig. 5 ▸ presents a schematic drawing of the structure of RUB-6. Atomic coordinates, displacement parameters and occupancy factors are listed in Table S3.

#### 3.3.3. Description of the structure   

The structure of RUB-6 consists of silicate layers [see Fig. 2 ▸(*a*)] possessing the topology of layer γ (see Sections 2.5 and 3.2.3). The layers of RUB-6 are not covalently bonded to each other. The shortest interlayer distance between the terminal oxygen atoms of two neighbouring layers is 3.5 Å (OH6–OH6). This excludes even the presence of substantial hydrogen bonds between the silanol/sil­oxy groups of neighbouring layers. Instead, intralayer hydrogen bonds exist between the oxygen atoms of terminal silanol/sil­oxy groups at a distance of 2.81 Å. Assuming that 4-amino­methyl-piperidine is included as a neutral molecule, the γ layers in RUB-6 have the composition [Si_40_O_76_(OH)_8_].

The intercalated 4-amino­methyl-piperidine molecule is disordered. While the positions of the 6-ring in all possible orientations of the molecule are almost identical, four partially occupied positions of the amino­methyl side-chain were detected from the remaining electron density and labelled N1, N2, C2 and C7 (see Fig. S7, Table S3). The shortest distances between the molecule and silicate layer (see Fig. S7) are *d*(N1⋯OH18) = 2.51 and *d*(N1⋯O21) = 2.53 Å. In addition, there are van der Waals contacts between carbon atoms and oxygen atoms of the layer, as well as between carbon atoms of neighbouring organic molecules in the *ab* plane. However, because of the disordered arrangement of the molecules within the structure, it is impossible to conclusively interpret these weak interactions.

According to the structure analysis and thermogravimetric analysis (TGA) (Marler *et al.*, 2020 ▸) the unit cell composition of RUB-6 was found to be [Si_40_O_76_(OH)_8_]·2C_6_H_14_N_2_. As already indicated by the TGA (Marler *et al.*, 2020 ▸), RUB-6 is free of molecular water. The organic molecule seems to adopt two different orientations between the silicate layers. These orientations lead to similar positions of the six-membered ring, with regards to the amino­methyl side-chain, however, pointing into two different directions. It was not possible to distinguish between the five carbon atoms and the nitro­gen atom in the six-membered ring (piperidine).

#### 3.3.4. Comparison with RUB-5   

The structures of RUB-6 and RUB-5 are closely related, having partially identical structures. The silicate layers of RUB-6 (layer γ) are terminated by silanol/sil­oxy groups and maintain a certain distance from each other.

Upon adding an additional silica layer δ [see Fig. 3 ▸(*a*)] in a formal condensation reaction with the silanol groups of layer γ, the α layer is generated. Interconnected α layers make up the complete structure of RUB-5. Consequently, RUB-5 may formally be considered as the interlayer expanded zeolite generated from the layered precursor of RUB-6 by adding additional SiO_4/2_ tetrahedra between the layers.

### 3.4. Disorder analysis of RUB-5   

#### 3.4.1. Exit wave reconstruction   

**Figure 6:**
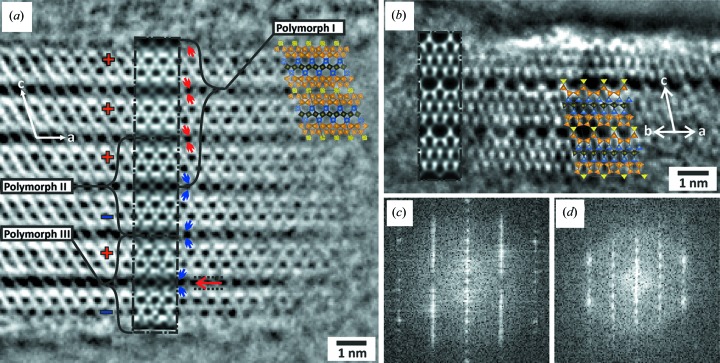
Reconstructed phases image viewed along the (*a*) [0−10] and (*b*) [−1−10] zone axes. (*a*) The irregular stacking of unit cells along the *c* axis is marked by + and − signs. Changes in the handedness of Si columns around gaps are marked by red and blue arrows, and the shifted (Δ*x* = −1/4, Δ*y* = +1/4) subunit is marked with black dotted lines and a red arrow. The black-bordered insets (dashed-dotted lines) are simulated images of RUB-5 polymorphs I, II and III in the [0−10] zone axis orientation. (*b*) Overlaid structure model and simulated image (dashed-dotted-border inset) of RUB-5 in the [−1−10] zone axis orientation. (*c*) Power spectra calculated from (*a*). (*d*) Power spectra calculated from (*b*).

In order to study the disorder from RUB-5, a phase image was reconstructed from a focal image series of a thin crystallite, with its [0−10] axis oriented parallel to the electron beam. After exit wave reconstruction, the following residual axial aberrations were minimized by numerical correction (Lehmann, 2000 ▸): focus *C*_1_, twofold astigmatism *A*_1_, second-order coma *B*_2_, threefold astigmatism *A*_2_ and third-order spherical aberration *C*_3_. The reconstructed structure projection image [Fig. 6 ▸(*a*)] shows an irregular stacking along the *c* axis with a thickness of about 105 Å that could not be directly seen from the diffraction data.

Starting from the top of the particle, the first part of the image can be described as a repetition of three α layers in the *c* direction. The stacking of α layers exclusively leads to polymorph I which is identical to the average structure of RUB-5, as already explained in the structural comparison of RUB-5 and RUB-6. The underlying part, however, is not a further repetition of layer α in the *c* direction. The fourth layer of the phase image can be properly described only by taking the α layer and flipping it 180° around the *a* axis with an additional shift of Δ*x* = −0.29 (generating layer β), which also results in a plausible chemical bonding with the third layer (see Fig. S8 for more details). A periodic repetition of αβαβαβ… (+−+−+−…) can be described as another polymorph (polymorph II) of RUB-5. Polymorph II has a base-centred lattice and the space group is *C*222_1_. The corresponding lattice constants relative to polymorph I can be calculated as
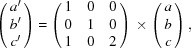
and result in *a*′ = 10.26, *b*′ = 10.64, *c*′ = 34.81 Å.

The following part of the experimental phase image along the stacking axis also shows a change in handedness as described above. Furthermore, a subunit of the basic structure [visualized in Fig. 6 ▸(*a*)] is shifted connecting the fifth and sixth parts. This element cannot be described by either a single unit cell of polymorph I (layer α) or polymorph II (layer α + β). In order to describe this region correctly, another polymorph was introduced by applying shifts for a specific layer sequence. A superstructure of polymorph I using two unit cells in the *c* direction was created. A shift vector of Δ*x* = −1/4, Δ*y* = +1/4 was applied to the structural part described by the sequence of LLBU *B* and *A* [see Fig. 3 ▸(*a*)]. The resulting superstructure correctly describes the last part of the experimental phase image with a chemically meaningful connection to the upper part. Applying a symmetry search for the constructed superstructure containing a layer shift resulted in a smaller lattice with triclinic symmetry. The lattice constants relative to polymorph I are determined as
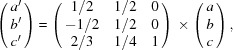
with *a*′ = 7.39, *b*′ = 7.39, *c*′ = 17.80 Å, α = 89.52, β = 78.05 and γ = 87.90°. This crystal structure is defined here as polymorph III of RUB-5. The corresponding power spectrum calculated from the phase image shown in Fig. 6 ▸(*a*) shows diffuse streaks along the c* axis as expected. All three polymorphs were used to perform multislice simulations. The overlay on the phase of exit wave [Fig. 6 ▸(*a*)] properly describes all stacking irregularities.

A second particle was found oriented with [110] parallel to the electron beam. The phase reconstruction and residual axial aberrations were handled as described above in this section. Fig. 6 ▸(*b*) shows the resulting reconstructed structure projection image. Along the stacking direction the particle is only about 5 nm-thick and has only a few unit cell repetitions along the [1−10] direction without construction errors of the framework. In spite of the small particle size, the structure can be confirmed with the overlaid structure model of polymorph I and the corresponding image simulation.

It should be noted here that polymorphs I and II are polytypic to each other, whereas polymorph III has no polytypic relation to the other polymorphs. As illustrated in Fig. 3 ▸(*a*) (LLBU *A* and LLBU *B*), the α layer could be divided into smaller layer units and thus other possible polymorphs could be described in a more systematic way by the order–disorder (OD) theory (Ferraris *et al.*, 2008 ▸). However, this is beyond the scope of this work and we are focused on the most prominent type of disorder (stacking disorder of polymorph I and polymorph II) with respect to the structure of the strongly related RUB-6. As already mentioned in Section 3.3.4, the α layer is simply a combination of layers δ + γ. Assuming that the precursor of both RUB-5 and RUB-6 forms γ layers in solution, it is likely that they either condense with an additional silica source (structurally layer δ) to RUB-5 or with suitable large organic molecules to RUB-6, thus forming four different stacking sequences, ++…, −−…, +−… and −+… In the case of RUB-5, this leads to either polymorph I or polymorph II defined here, with two different enantiomorphs for each of them. The only possible shift between two layers is Δ*x* = ±1/2, Δ*y* = ±1/2. However, due to the base-centering, this does not lead to a crystallographic change in the structure. Moreover, the disorder present in RUB-6 could be reasonably described in a similar way as for RUB-5. Unfortunately, it was not possible to obtain HRTEM images of RUB-6 due to its much higher beam sensitivity.

#### 3.4.2. Disorder modelling and diffraction simulations   

The different polymorphs or the stacking of different layers derived from the structural image of RUB-5 produced by exit wave reconstruction [Fig. 6 ▸(*a*)] are examined in this section. This is a good opportunity to compare diffuse scattering between the Bragg reflections measured by 3D ED with diffuse scattering calculated from disordered crystals. The crystals measured with 3D ED were significantly thicker and thus contain more stacking sequences than the observed stacking sequences in the single structural image presented in the previous section. If the essential features of the diffuse scattering can be described with the stacking sequences derived from the structural image, it can be taken as good validation for the most frequent stacking faults. The program *DISCUS* was used to model RUB-5 superstructures based on LLBUs and to compare the simulated and experimental electron diffraction patterns, in analogy to our previous work (Krysiak *et al.*, 2018 ▸).

It should be considered that the interpretation of thermal analysis (DTA and TGA) and the ^29^Si-^1^H CP MAS NMR spectrum of RUB-5 (Marler *et al.*, 2020 ▸) results in a small number of silanol (Si—OH) defects, possibly due to unlinked or partially unlinked silica γ layers, like in the structure of RUB-6. Whenever an unconnected γ layer is present in the stacking sequence of RUB-5, a local change in the *d*-spacing is expected, which is not the case for the stacking sequences α + α, β + β, α + β and β + α (all stacking events used for the modelling are listed in Table 2 ▸). It should be noted here that organic molecules and/or water in the interlayer region of unlinked silica γ layers are ignored for the further discussion, modelling and simulations. So, the detected diffraction data would be expected to show diffuse scattering along the 00*l* reflections due to a non-periodic length of the *c* axis.

As mentioned above, the limitations of the goniometer stage and specimen holder geometry combined with the preferred orientation of the crystals inhibited the measurement of the 00*l* reflections. Few crystals were found whose diffraction pattern shows 00*l* reflections, but only one crystal was closely oriented to the zone axis [110]. The crystal was measured without electron beam precession from −20 to +10° in 1° steps. Due to the low beam stability of the material in this rare crystal orientation, it was not possible to measure a larger tilt interval. Nevertheless, the integrated zone image (Fig. S9) was reconstructed with the Matlab-based script *diffuse_extractor* (Kolb *et al.*, 2019 ▸) and the extracted zone shows diffuse scattering along the 00*l* reflections.

In order to roughly estimate the number of unlinked γ layers, the disorder modelling in *DISCUS* was, in a first step, carried out only as a function of the stacking probability for the α and γ layers. The determination of the absolute amount of layer γ (*p*_*y*_) (the lower correlation matrix listed in Table 3 ▸) was used to avoid all stacking events apart from α + γ and γ + α. The 00*l* reflections (*l* from 2.5 to 6.5) were chosen for a comparison of experimental and simulated diffuse scattering using the script *diffuse_compare* (Kolb *et al.*, 2019 ▸). Whenever an α or γ layer is stacked on a γ layer, the stacking distance is increased (see Table 2 ▸) due to the missing connection through the δ layers. Instead we postulate that organic molecules and/or water are present in the interlayer region, like in RUB-6. This means that, if only γ layers are stacked on top of each other (*p*_*y*_ = 1.0), the structure of RUB-6 is built, if the interlayer region is neglected. For simplification, the organic molecules and/or water are not taken into account for the modelling and diffraction simulations. The highest similarity between the experimental and simulated diffuse rod converged for *p*_*y*_ = 0.05 (3) (see Fig. S9). This value was then used for further simulations in which all stacking sequences (listed in Table 2 ▸) were taken into account. It is interesting that the pattern of the diffuse scattering on the entire simulated [110] zone is similar to the experimental one. It should also be emphasized that any stacking of only α and β layers (*p*_*y*_ = 0.0) means that no diffuse scattering is to be expected on the [110] zone, because the structure projection along [110] is not affected by the stacking faults. In addition to the temperature-dependent XRPD measurements, results from DTA-TGA and NMR, the hypothesis of partially or completely unlinked silicate layers is supported (Marler *et al.*, 2020 ▸).

**Figure 7:**
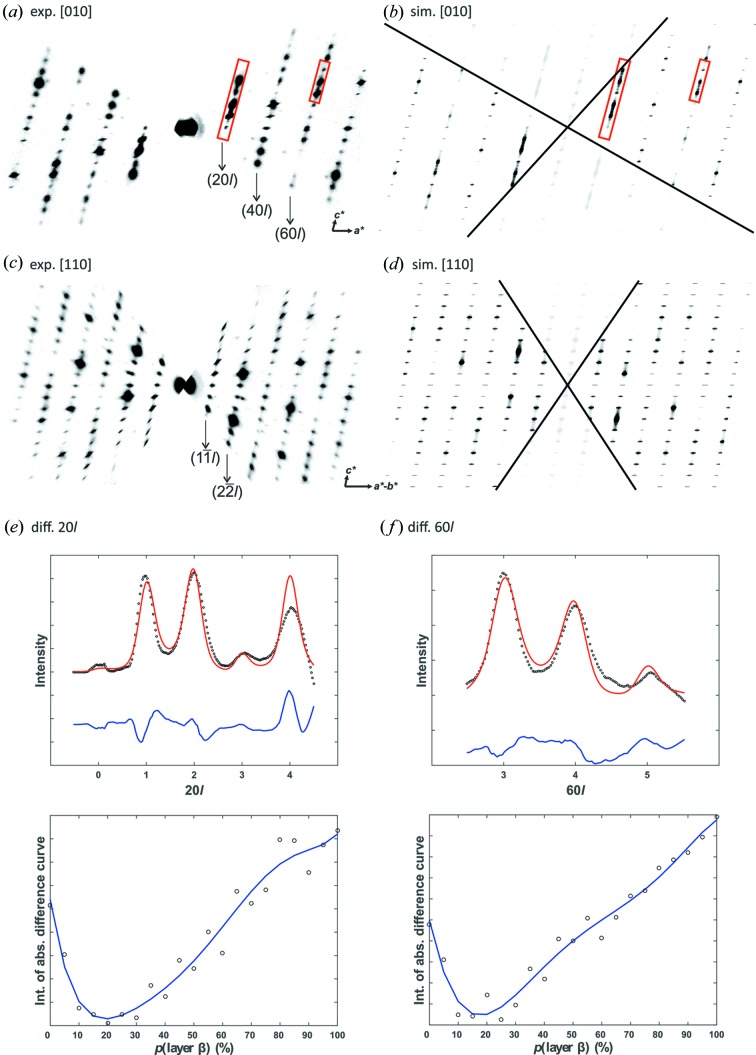
Comparison of experimental and simulated (*p*_*x*_ = 0.225, *p*_*y*_ = 0.05) electron diffraction patterns of zones (*a*) and (*b*) [010], and (*c*) and (*d*) [110]. Intensity profiles taken along the corresponding diffraction lines are marked in (*a*) and (*b*) by red rectangles. Plot of experimental (black circles) and the simulated (red) line profiles (*e*) 20*l* and (*f*) 60*l* for the stacking probabilities *p*_*x*_ = 0.20 and *p*_*x*_ = 0.25, respectively. Plot of the integrated absolute difference between simulated and experimental line profiles against the stacking probability of layer β for line profiles (*e*) 20*l* and (*f*) 60*l*. Blue trend lines were fitted by a polynomial degree of six.

Although ADT scans a large volume of reciprocal space, tilt steps of 1°, even 0.5°, are not sufficient to resolve the diffuse scattering between Bragg reflections for a quantitative analysis. For the following disorder analysis, we used our newly developed Fast-ADT technique, which is based on a continuous tilt acquisition, in order to measure diffuse scattering more accurately (Plana-Ruiz *et al.*, 2020 ▸). Thus, even 3D ED data taken from oriented crystals can be used for quantitative disorder analysis. Examples of integrated zone images {[010] and [110]} based on Fast-ADT data are shown in Fig. 7 ▸.

The input parameter for the stacking module of *DISCUS* is the correlation matrix which defines the probability for a specific sequence of layer types as a function of the stacking probabilities *p*_*x*_ and *p*_*y*_ (Table 3 ▸). The probabilities for the presence of layer β, α and γ are *p*_*x*_, (1 − *p*_*x*_) and *p*_*y*_, respectively. The corresponding simulated ED patterns were calculated for *p*_*x*_ = 0.0 to 1.0 in 0.05 steps while keeping *p*_*y*_ fixed at 0.05. Qualitatively, the experimental and simulated patterns (*p*_*x*_ = 0.225, *p*_*y*_ = 0.05) of diffuse scattering on the crystallographic zones are comparable (Fig. 7 ▸). The strongest diffuse streaks of zone [010] are observed for 20*l* and 60*l* reflections. These diffuse streaks were taken into account for a quantitative disorder analysis dependent on the stacking probability of layer β. Therefore, the extracted experimental diffuse streaks were compared with the diffuse lines calculated in *DISCUS* by *diffuse_compare*. The best agreement between the experimental and simulated diffuse lines could be achieved for *p*_*x*_ = 0.20 (5) and *p*_*y*_ = 0.05 in the case of 20*l* reflections and *p*_*x*_ = 0.25 (5) and *p*_*y*_ = 0.05 for the 60*l* reflections. The given uncertainties should be considered as rough estimates. Inelastic scattering, an insufficiently sensitive detector and thus difficulties in the background correction do not allow a more precise disorder analysis of this material yet (Kolb *et al.*, 2019 ▸). It is worth mentioning that polymorph III has not been taken into account for the disorder simulations presented here, since these subtleties are not yet distinguishable from 3D ED data at the time of writing.

However, to further emphasize the close structural relationship between RUB-5 and RUB-6, integrated zone images {[010] and [110]} of RUB-6 and RUB-5 were compared. As shown in Fig. S10, the patterns of the diffuse scattering in both zones closely resemble each other; this supports the hypothesis that the formation of RUB-5 and RUB-6 occurs through a similar intermediate step (Marler *et al.*, 2020 ▸).

## 4. Conclusions   

In this work, we demonstrate the structure determination of the new hydrous layer silicate RUB-6 and its related zeolite, RUB-5. The latter represents a new silica polymorph with a unique framework type. As described in a recent paper (Krysiak *et al.*, 2018 ▸), stacking disorder and intergrowth of different polymorphs within single nanocrystals generally requires a combination of 3D ED, exit wave reconstruction, structure modelling and diffraction simulation in order to obtain a comprehensive structure description.

The average structures of RUB-5 and RUB-6 were solved *ab initio* based on ADT data. All silicon and oxygen atoms of the framework could be directly located by direct methods. The ADT method again proved to be a very valuable tool to determine the structure of nanometre-sized crystals, which possess a complex disordered structure with 32 atoms in the asymmetric unit.

RUB-6 and RUB-5 are built up by the same LLBUs (γ layers). RUB-6 can be described as a hydrous layer silicate containing 4-amino­methyl-piperidine inside the interlayer region. In the case of RUB-5, these layers are connected through an additional silicon to a new type of zeolite. Both structures form crystals with the same space group *C*2 and a similar lattice. The contraction of the unit cell along the stacking *c* axis by about 2.4 Å (from RUB-6 to RUB-5) corresponds to the formation of an interconnection between the silicate layers. It is also interesting that the structural motif of RUB-5 resembles a quartz-like topology (LLBU A) together with a porous LLBU B of unique topology.

The diffuse scattering observed in the experimental reciprocal space was explained successfully by means of a disorder model corresponding to the atomically resolved structure images generated by the exit wave reconstruction. The diffuse scattering is thus caused by intergrowth of at least two different polytypes. For RUB-5, superstructures including stacking disorder could be modelled. By comparing their respective simulated electron diffraction space with the experimental data, a description of the stacking disorder could be obtained. In addition, the extracted diffuse scattering was used to show the relationship between the formation process of RUB-5 and RUB-6. The comparable pattern of the diffuse scattering in both phases leads to the conclusion that the formation of RUB-5 and RUB-6 occurs through a similar intermediate step. Our results suggest that the disorder in RUB-5/6 is inherited from the disorder of a common intermediate phase.

The application of electron diffraction tomography allowed us to solve a long-existing mystery in the structure analysis of layered silicates which was not accessible through XRPD. Moreover, a new topology in the crystal structures of the hydrous layer silicate RUB-6 and the related zeolite RUB-5 has been revealed and opened the door to the synthesis of novel catalytically active zeolites. A comprehensive structural study of new functionalized materials with crystal disorder, using the method presented here, may solve many important material-related questions in the near future.

## Supplementary Material

Crystal structure: contains datablock(s) RUB-5, RUB-6. DOI: 10.1107/S2052252520003991/fc5040sup1.cif

Supporting information file. DOI: 10.1107/S2052252520003991/fc5040sup2.pdf

CCDC references: 1991689, 1995979

## Figures and Tables

**Table 1:** Crystallographic information about ADT measurements and structure solutions of RUB-5 and RUB-6 Lattice parameters were taken from the Rietveld refinements.

System	RUB-5	RUB-6
Tilt range (°)	−60/+60	−60/+60
No. of sampled reflections	7994	5459
No. of independent reflections	1641	982
Resolution (Å)	0.8	1.0
Completeness (%)	81	82
*R*_sym_	0.167	0.132
Overall *U* (Å^2^)	0.035	0.049
Residual *R*_F_ (*SIR*2014)	0.171	0.141
Reflection to parameter ratio	7.9	4.7
No. of independent Si and O atoms	33	32
Space group	*C*2	*C*2
*a* (Å)	10.2676 (6)	10.1226 (22)
*b* (Å)	10.6449 (6)	10.6694 (26)
*c* (Å)	18.1558 (6)	20.5528 (24)
α (°)	90.0	90.0
β (°)	106.36 (2)	105.86 (1)
γ (°)	90.0	90.0
*V* (Å^3^)	1906.0 (2)	2135.2 (7)

**Table 2:** List of stacking events used for disorder modelling in *DISCUS* and the corresponding interpretation of the local structure

Event	Δ*x*	Δ*y*	Δ*z*	*P*_*ij*_	Interpretation
α + α	0	0	1	(1 − *p*_*x*_)(1 − *p*_*y*_)	Polymorph I
α + β	0	0	1	*p*_*x*_(1 − *p*_*y*_)	Polymorph II
α + γ	0	0	1.120	*p*_*y*_	Like RUB-6
β + α	0	0	1	*p*_*x*_(1 − *p*_*y*_)	Polymorph II
β + β	0	0	1	(1 − *p*_*x*_)(1 − *p*_*y*_)	Polymorph I
β + γ	0	0	1.120	*p*_*y*_	Like RUB-6
γ + α	0	0	1	(1 − *p*_*y*_)/2	Polymorph I
γ + β	0	0	1	(1 − *p*_*y*_)/2	Polymorph II
γ + γ	0	0	1.120	*p*_*y*_	Like RUB-6

**Table:** The lower correlation matrix was used to estimate *p*_*y*_.

All layers	α	β	γ
α	(1 − *p*_*x*_)(1 − *p*_*y*_)	*p*_*x*_(1 − *p*_*y*_)	*p*_*y*_
β	*p*_*x*_(1 − *p*_*y*_)	(1 − *p*_*x*_)(1 − *p*_*y*_)	*p*_*y*_
γ	(1 − *p*_*y*_)/2	(1 − *p*_*y*_)/2	*p*_*y*_

**Table:** 

Layer γ	α	β	γ
α	(1 − *p*_*y*_)	0	*p*_*y*_
β	0	0	0
γ	(1 − *p*_*y*_)	0	*p*_*y*_
