# Supplementary material for: New zeolite-like RUB-5 and its related hydrous layer silicate RUB-6 structurally characterized by electron microscopy
Source: IUCrJ. 2020 Apr 21;7(Pt 3):522–34. doi: 10.1107/S2052252520003991 (PMC7201290; doi:10.1107/S2052252520003991)
Supplement: Supplementary file 2 [file m-07-00522-sup2.pdf]

IUCrJ

Volume 7 (2020)

Supporting information for article:

New zeolite-like RUB-5 and its related hydrous layer silicate RUB-6 structurally characterised by electron microscopy

Yaşar Krysiak, Bernd Marler, Bastian Barton, Sergi Plana-Ruiz, Hermann Gies, Reinhard B. Neder and Ute Kolb

S1. Crystal images

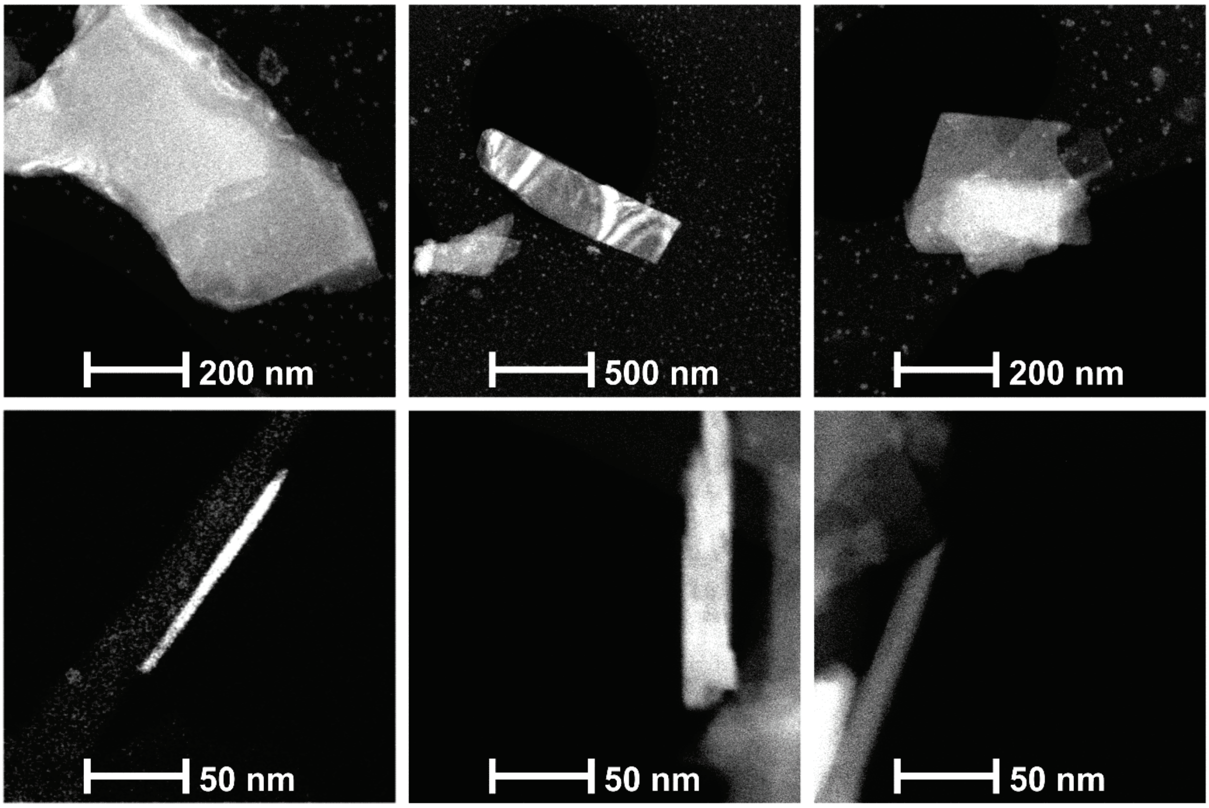

Figure S1 µm-probe STEM images of RUB-5 collected with low electron dose (C2 of 10µm, SS8 and GL8) showing the plate-like morphology (upper part). The plate thickness of the crystals is shown (lower part) by upstanding crystals at the holes of the carbon film.

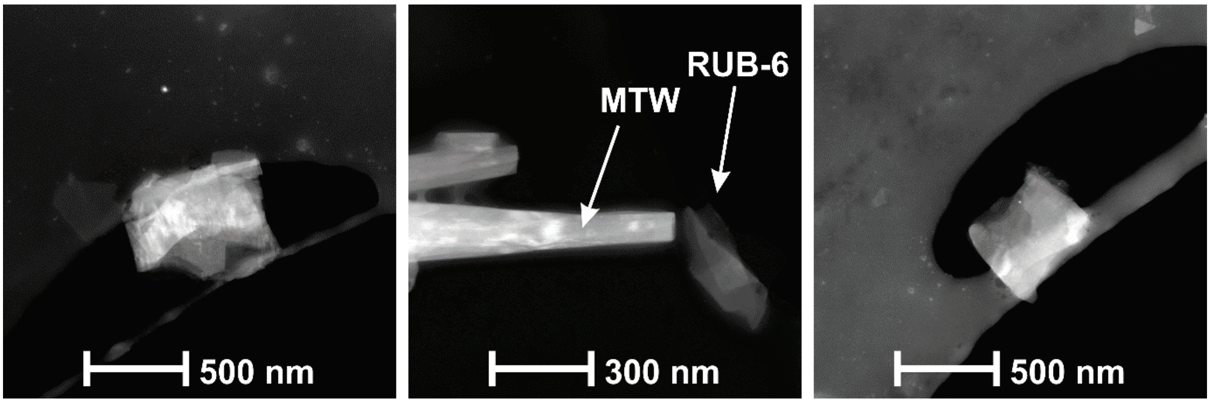

Figure S2 µm-probe STEM images of RUB-6 showing highly agglomerated plate-like crystals. Images are recorded with much higher electron dose (C2 of 50µm, SS8 and GL8) than for RUB-5 because of low scattering power of RUB-6. Zeolite MTW in comparison with RUB-6 (middle).

S2. Energy-dispersive X-ray spectroscopy (EDXS)

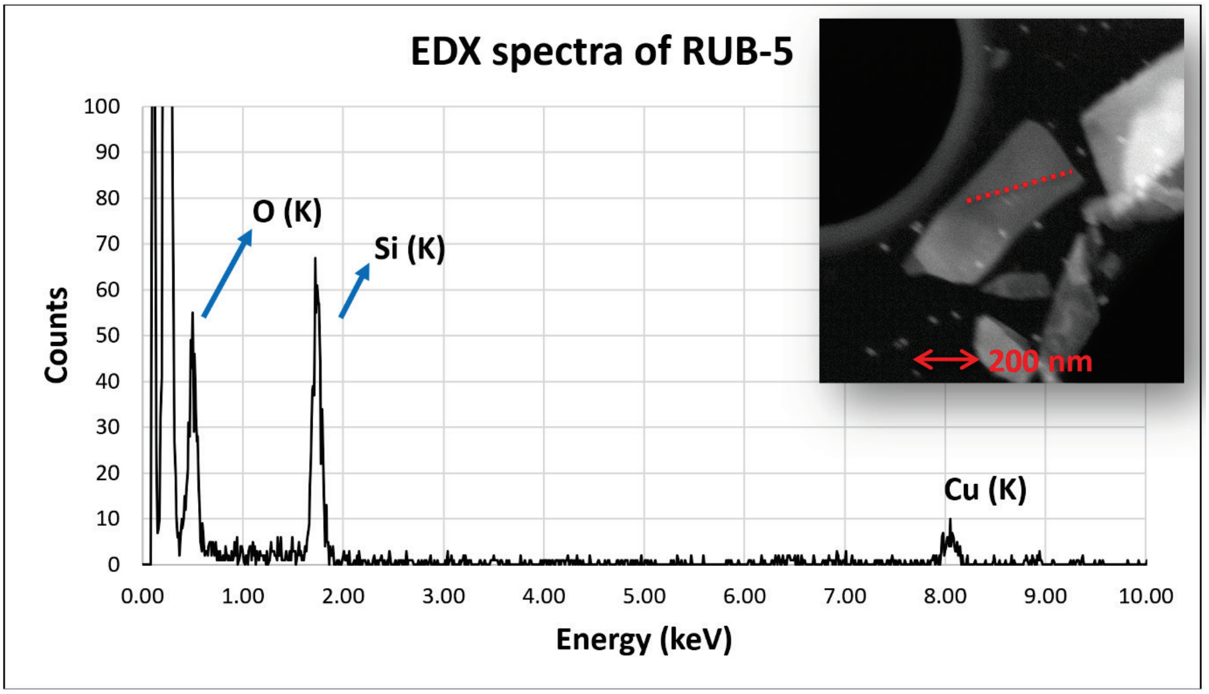

Figure S3 EDX spectrum of RUB-5 particle shown in STEM image with the most intense fluorescence peaks labelled. The Cu K peak at about 8 keV is caused by the TEM Grid.

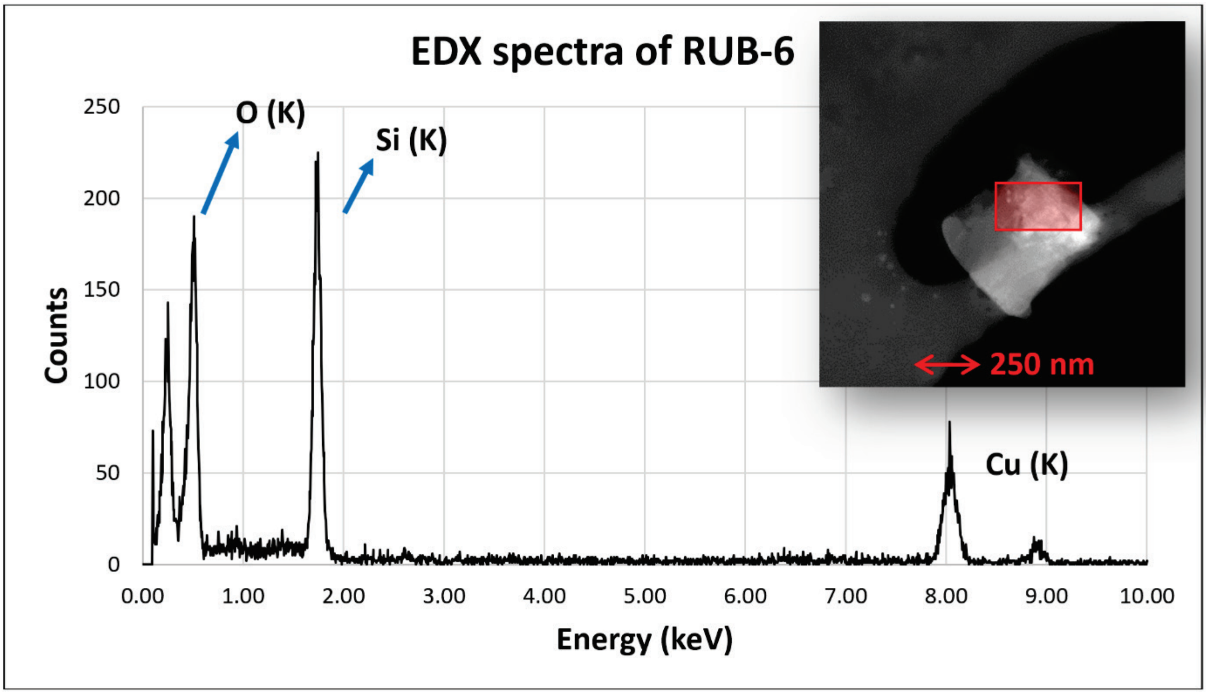

Figure S4 EDX spectrum of RUB-6 particle shown in STEM image with the most intense fluorescence peaks labelled. The Cu K peak at about 8 keV is caused by the TEM Grid.

S3. cif data determined by ADT

S3.1. RUB-5

```
######################################################################
#
# Structure solution of RUB-5
#
######################################################################

data_I
_symmetry_cell_setting           monoclinic
_symmetry_space_group_name_H-M   'C 2'
_symmetry_Int_Tables_number      5
_space_group_name_Hall           'C 2y'
loop_
_symmetry_equiv_pos_site_id
_symmetry_equiv_pos_as_xyz
1 x,y,z
2 -x,y,-z
3 1/2+x,1/2+y,z
4 1/2-x,1/2+y,-z
_cell_length_a                   10.27
_cell_length_b                   10.64
_cell_length_c                   18.16
_cell_angle_alpha                90
_cell_angle_beta                 106.4
_cell_angle_gamma                90
_cell_volume                     1903.66
loop_
 _atom_site_label
 _atom_site_type_symbol
 _atom_site_fract_x
 _atom_site_fract_y
 _atom_site_fract_z
 _atom_site_adp_type
 _atom_site_U_iso_or_equiv
 _atom_site_site_symmetry_multiplicity
 _atom_site_occupancy
 _atom_site_calc_flag
 _atom_site_refinement_flags
 _atom_site_disorder_assembly
 _atom_site_disorder_group
  Si3 Si 0.05086 0.73442 0.37159 Uiso 0.0149 4 1 d . . .
  Si4 Si 0.11773 0.06626 0.23936 Uiso 0.0163 4 1 d . . .
  Si5 Si 0.24467 0.88933 0.49431 Uiso 0.0322 4 1 d . . .
  Si6 Si 0.34768 0.87124 0.26103 Uiso 0.0136 4 1 d . . .
  Si7 Si 0.0699 0.24157 0.38396 Uiso 0.0432 4 1 d . . .
  Si8 Si 0.41852 0.02643 0.1267 Uiso 0.0199 4 1 d . . .
  Si10 Si 0.86695 0.91446 0.24052 Uiso 0.0214 4 1 d . . .
  Si11 Si 0.70398 0.71956 0.12492 Uiso 0.0445 4 1 d . . .
  Si12 Si 0.17398 0.63515 0.24603 Uiso 0.0227 4 1 d . . .
  O1 O 0.60872 0.7714 0.05309 Uiso 0.014 4 1 d . . .
  O2 O 0.2653 0.98759 0.27036 Uiso 0.0229 4 1 d . . .
  O3 O 0.17745 0.82455 0.41151 Uiso 0.0268 4 1 d . . .
  O4 O 0.93347 0.81916 0.32738 Uiso 0.0076 4 1 d . . .
  O5 O 0.13201 0.48988 0.51922 Uiso 0.0205 4 1 d . . .
  O6 O 0.00724 0.98399 0.23391 Uiso 0.014 4 1 d . . .
  O7 O 0.19767 0.28974 0.43321 Uiso 0.0185 4 1 d . . .
  O8 O 0.12711 0.98766 0.50161 Uiso 0.0309 4 1 d . . .
  O9 O 0.98182 0.16435 0.42375 Uiso 0.026 4 1 d . . .
  O10 O 0.99598 0.65914 0.43109 Uiso 0.0287 4 1 d . . .
  O11 O 0.42422 0.94737 0.05741 Uiso 0.0229 4 1 d . . .
  O12 O 0.24451 0.74456 0.23128 Uiso 0.0177 4 1 d . . .
  O13 O 0.12557 0.15084 0.3308 Uiso 0.0484 4 1 d . . .
  O14 O 0.1142 0.64519 0.31917 Uiso 0.036 4 1 d . . .
  O15 O 0.29076 0.51476 0.26016 Uiso 0.0333 4 1 d . . .
  O16 O 0.43172 0.8991 0.19032 Uiso 0.0261 4 1 d . . .
  O17 O 0.78397 0.84264 0.16766 Uiso 0.0312 4 1 d . . .
  O19 O 0.98517 0.36567 0.34481 Uiso 0.0617 4 1 d . . .
```

```
  O20 O 0.12415 0.14401 0.19001 Uiso 0.0122 4 1 d . . .
  O28 O 0.33 0.13371 0.11661 Uiso 0.0299 4 1 d . . .
  O O 0.44333 0.08472 0.82495 Uiso 0.0253 4 1 d . . .
  Si1 Si 0 0.06799 0.5 Uiso 0.0027 2 1 d . . .
  Si2 Si 0 0.57305 0.5 Uiso 0.024 2 1 d . . .
  Si9 Si 0 0.34734 0 Uiso 0.0295 2 1 d . . .


#END
```

S3.2. RUB-6

```
######################################################################
#
# Structure solution of RUB-6
#
######################################################################

data_I
_symmetry_cell_setting           monoclinic
_symmetry_space_group_name_H-M   'C 2'
_symmetry_Int_Tables_number      5
_space_group_name_Hall           'C 2y'
loop_
_symmetry_equiv_pos_site_id
_symmetry_equiv_pos_as_xyz
1 x,y,z
2 -x,y,-z
3 1/2+x,1/2+y,z
4 1/2-x,1/2+y,-z
_cell_length_a                   10.13
_cell_length_b                   10.67
_cell_length_c                   20.56
_cell_angle_alpha                90
_cell_angle_beta                 105.9
_cell_angle_gamma                90
_cell_volume                     2137.25
loop_
 _atom_site_label
 _atom_site_type_symbol
 _atom_site_fract_x
 _atom_site_fract_y
 _atom_site_fract_z
 _atom_site_adp_type
 _atom_site_U_iso_or_equiv
 _atom_site_site_symmetry_multiplicity
 _atom_site_occupancy
 _atom_site_calc_flag
 _atom_site_refinement_flags
 _atom_site_disorder_assembly
 _atom_site_disorder_group
  Si1 Si 0.56515 0.23137 0.38894 Uiso 0.0359 4 1 d . . .
  Si2 Si 0.73917 0.40083 0.49356 Uiso 0.0303 4 1 d . . .
  Si3 Si 0.90769 0.22518 0.60691 Uiso 0.05 4 1 d . . .
  Si4 Si 0.40077 0.40404 0.28369 Uiso 0.0289 4 1 d . . .
  Si7 Si 0.84568 0.04209 0.71095 Uiso 0.0521 4 1 d . . .
  Si8 Si 0.77085 0.20016 0.8277 Uiso 0.0741 4 1 d . . .
  O1 Si 0.62541 0.8532 0.70562 Uiso 0.0712 4 1 d . . .
  Si9 O 0.80566 0.29063 0.56111 Uiso 0.1058 4 1 d . . .
  O2 O 0.64163 0.15669 0.34298 Uiso 0.0712 4 1 d . . .
  O3 O 0.66911 0.31335 0.43703 Uiso 0.0686 4 1 d . . .
  Si10 Si 0.55301 0.01431 0.83029 Uiso 0.0712 4 1 d . . .
  O4 O 0.45207 0.29661 0.3458 Uiso 0.0986 4 1 d . . .
  Si11 O 0.49643 0.12072 0.42744 Uiso 0.0712 4 1 d . . .
  O5 O 0.00699 0.30576 0.64432 Uiso 0.0704 4 1 d . . .
  O6 Si 0.79916 0.62699 0.72218 Uiso 0.022 4 1 d . . .
  O7 O 0.85945 0.13516 0.65709 Uiso 0.1222 4 1 d . . .
  O8 O 0.31219 0.4956 0.29747 Uiso 0.0423 4 1 d . . .
```

```
 O9 O 0.01394 0.1474 0.57579 Uiso 0.0591 4 1 d . . .
 O10 O 0.52766 0.45688 0.26776 Uiso 0.047 4 1 d . . .
 O11 O 0.71289 0.96727 0.68647 Uiso 0.0513 4 1 d . . .
 O12 O 0.62647 0.49684 0.50294 Uiso 0.0813 4 1 d . . .
 O13 O 0.57938 0.8973 0.78969 Uiso 0.0519 4 1 d . . .
 O14 O 0.83906 0.1436 0.7743 Uiso 0.0751 4 1 d . . .
 O15 O 0.87485 0.47005 0.50163 Uiso 0.083 4 1 d . . .
 O16 O 0.72079 0.72199 0.72736 Uiso 0.0975 4 1 d . . .
 O17 O 0.6774 0.05979 0.83937 Uiso 0.0933 4 1 d . . .
 O18 O 0.90249 0.27822 0.90013 Uiso 0.1387 4 1 d . . .
 O19 O 0.29128 0.34137 0.22564 Uiso 0.1015 4 1 d . . .
 O21 O 0.50157 0.94996 0.89416 Uiso 0.0141 4 1 d . . .
 O23 O 0.40622 0.06345 0.78849 Uiso 0.0742 4 1 d . . .
 Si5 Si 0.5 0.05318 0.5 Uiso 0.0417 2 1 d . . .
 Si6 Si 0 0.07144 0.5 Uiso 0.0524 2 1 d . . .

#END
```

S4. Rietveld refinements

S4.1. Rietveld plots

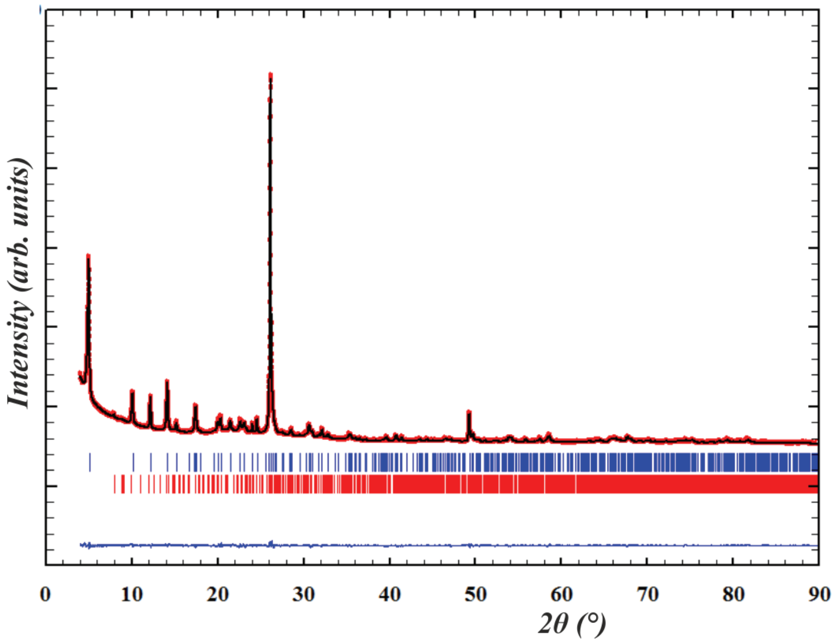

Figure S5 Plot of the diffraction patterns after Rietveld analysis of RUB-5. Experimental (red) and calculated (black) patterns are shown above the difference plot (blue line). Tick marks for allowed reflections (blue = RUB-5, red = ZSM-5) are given. Intensities in arbitrary units.

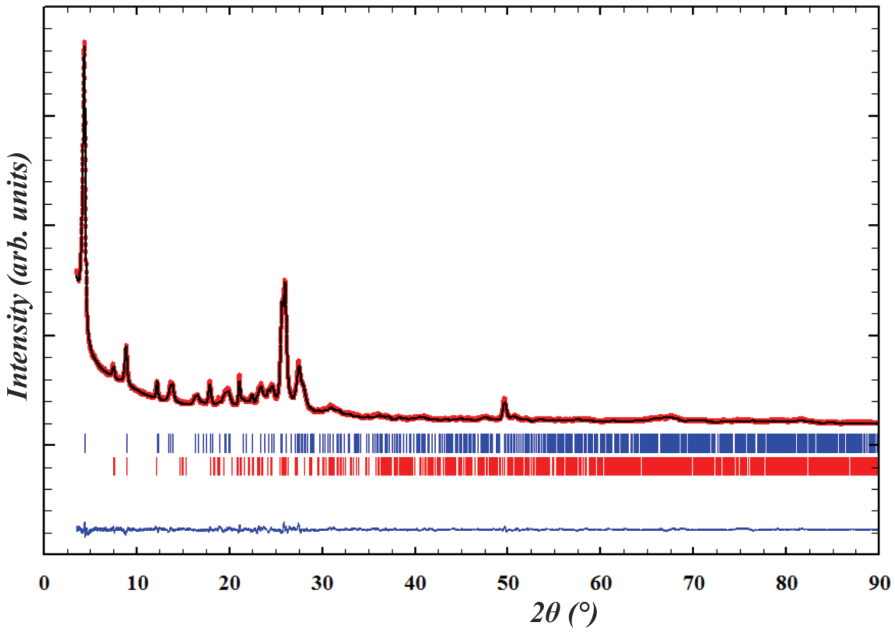

Figure S6 Plot of the diffraction patterns after Rietveld analysis of RUB-6. Experimental (red) and calculated (black) patterns are shown above the difference plot (blue line). Tick marks for allowed reflections (blue = RUB-6, red = ZSM-12) are given. Intensities in arbitrary units.

S4.2. Parameters for the Rietveld refinements

Table S1 Experimental and crystallographic parameters for the Rietveld refinements of zeolite RUB-5 and layer silicate RUB-6.

	RUB-5	RUB-6
Diffractometer	Siemens D5000 with 6° Braun PSD	
Wavelength	1.54060 Å	
Sample	powder in 0.3 mm glass capillary	
2θ range (°)	4.0 - 90.0	3.5 - 90.0
Step size in 2θ (°)	0.007866	0.007888
No. steps	11387	11007
No. contributing reflections	833	928
No. geometric restraints	129	135
No. structural parameters	102	113
No. profile parameters	21	21
FWHM at ca. 25° 2θ (°)	0.12 - 0.32	0.22 - 0.82
Spherical harmonics component Y00	0.194598	5.397610
Spherical harmonics component Y20	-0.977645	-0.950710

Spherical harmonics component Y22+	1.146215	2.325654
Spherical harmonics component Y22-	-0.702390	-1.144377
Spherical harmonics component Y40	-0.079124	-0.555880
Spherical harmonics component Y42+	1.086668	5.505152
Spherical harmonics component Y42-	-1.325602	1.866171
Spherical harmonics component Y44+	-0.742849	-1.067283
Impurity in weight percent	1.6(2)% of ZSM-5	6.7(3)% of ZSM-12
R_{Bragg}	0.033	0.026
R_F	0.031	0.025
R_{wp}	0.086	0.089
R_{exp}	0.048	0.047
$\chi 2$	3.3	3.7
Space group	*C2* (No. 5)	*C2* (No. 5)
a_0 (Å)	10.2703 (6)	10.1250 (24)
b_0 (Å)	10.6423 (6)	10.6669 (26)
c_0 (Å)	18.1565 (6)	20.5551 (38)
β (°)	106.35 (2)	105.86 (1)
V_{UC} (Å^3)	1904.3 (2)	2135 (2)
Calculated density (g/cm^3)	2.20	2.01
Unit cell content	[$Si_{42}O_{84}$]	[$Si_{40}O_{76}(OH)_8$] * $2C_6H_{14}N_2$

S4.3. Schematic drawing of the structure RUB-6

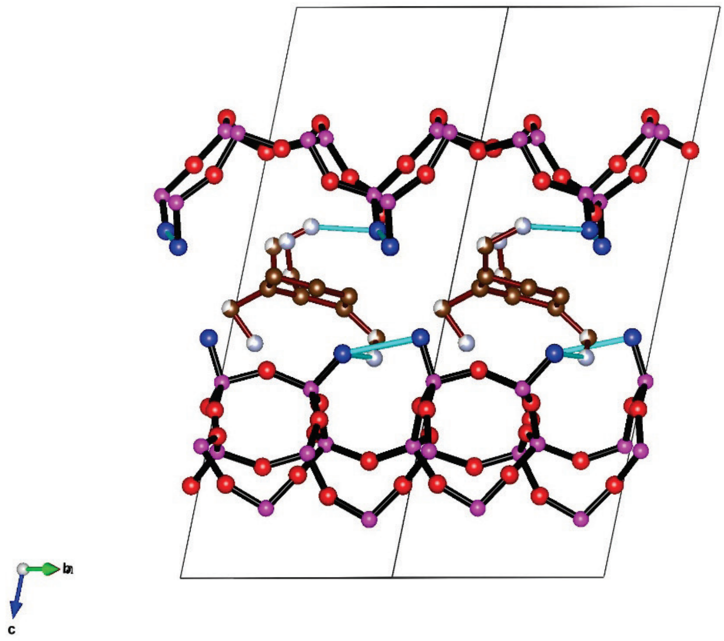

Figure S7 Section of the structure of RUB-6 showing the position of the organic molecule/cation in the interlayer region (projection along [-110]) (purple = Si, red = O, blue = terminal OH groups, brown = C, gray = N). Blue lines = hydrogen bonds.

S4.4. Atomic coordinates

Table S2 Atomic coordinates, displacement parameters and occupancy factors of the average structure of RUB-5.

Atom	x	y	z	U_{iso} (Å²)	Occ.
SI1	0.50000	0.1615(22)	0.50000	0.012	1.00000
SI2	0.4285(15)	0.0102(15)	0.6263(11)	0.012	1.00000
SI3	0.00000	0.9418(22)	0.00000	0.012	1.00000
SI4	0.2519(15)	0.6131(15)	0.0095(7)	0.012	1.00000
SI5	0.2839(15)	0.2815(15)	0.3717(7)	0.012	1.00000
SI6	0.00000	0.4412(22)	0.00000	0.012	1.00000
SI7	0.1496(15)	0.6396(15)	0.2507(7)	0.012	1.00000
SI8	0.0638(19)	0.7751(15)	0.1203(7)	0.012	1.00000
SI9	0.3708(15)	0.4327(15)	0.2502(11)	0.012	1.00000
SI10	0.0562(15)	0.2742(15)	0.1332(7)	0.012	1.00000
SI11	0.1185(15)	0.1017(15)	0.2532(11)	0.012	1.00000
SI12	0.6726(15)	0.1236(15)	0.7417(7)	0.012	1.00000
O1	0.2314(34)	0.5176(37)	0.2314(22)	0.024	1.00000
O2	0.1856(37)	0.1879(45)	0.0880(19)	0.024	1.00000
O3	0.3811(45)	0.3600(41)	0.1774(19)	0.024	1.00000
O4	0.0180(37)	0.8581(45)	0.0739(19)	0.024	1.00000
O5	0.4963(37)	0.5285(45)	0.2725(22)	0.024	1.00000
O6	0.3753(41)	0.2452(49)	0.4553(15)	0.024	1.00000
O7	0.0692(34)	0.1985(45)	0.1860(22)	0.024	1.00000
O8	0.0069(49)	0.3584(45)	0.0730(19)	0.024	1.00000
O9	0.1051(45)	0.3602(37)	0.1919(19)	0.024	1.00000
O10	0.1830(37)	0.7188(34)	0.0504(19)	0.024	1.00000

O11	0.2573(52)	0.7527(37)	0.2843(19)	0.024	1.00000
O12	0.1853(45)	0.1688(37)	0.3334(19)	0.024	1.00000
O13	0.0362(41)	0.6739(37)	0.1748(19)	0.024	1.00000
O14	0.3687(37)	0.3389(37)	0.3180(19)	0.024	1.00000
O15	0.1375(37)	0.5277(45)	0.0302(19)	0.024	1.00000
O16	0.1373(37)	1.0243(49)	0.0160(19)	0.024	1.00000
O17	0.2317(41)	0.0033(30)	0.2378(19)	0.024	1.00000
O18	0.4672(56)	0.0674(41)	0.5612(19)	0.024	1.00000
O19	0.1970(41)	0.3925(37)	0.3983(22)	0.024	1.00000
O20	0.0800(41)	0.5970(34)	0.3143(19)	0.024	1.00000
O21	0.5661(37)	0.0887(45)	0.6625(15)	0.024	1.00000
OH1	0.363(11)	0.484(11)	0.5296(56)	0.024	0.562(9)
OH2	0.50000	0.5840(64)	0.50000	0.024	1.00000
OH3	0.1820(64)	0.0358(64)	0.1282(30)	0.024	1.00000

Note: Realistic s.u. were obtained by multiplying the calculated s.u. with a factor of 3.74 (as provided by the FullProf program after the final stage of the Rietveld refinement).

Table S3 Atomic coordinates, displacement parameters and occupancy factors of the average structure of RUB-6.

Atom	x	y	z	U_{iso} (Å^2)	Occ.
SI2	0.4437(23)	0.0091(19)	0.6690(9)	0.012	1.00000
SI3	0.00000	0.9459(28)	0.00000	0.012	1.00000
SI4	0.2614(23)	0.6257(19)	0.0153(9)	0.012	1.00000
SI5	0.3050(23)	0.3142(19)	0.3415(9)	0.012	1.00000
SI6	0.00000	0.4457(28)	0.00000	0.012	1.00000
SI7	0.1508(23)	0.6319(23)	0.2169(9)	0.012	1.00000
SI8	0.0631(23)	0.7720(19)	0.1026(9)	0.012	1.00000
SI9	0.3929(23)	0.4494(19)	0.2207(9)	0.012	1.00000
SI10	0.0428(23)	0.2946(19)	0.1209(9)	0.012	1.00000
SI11	0.1444(23)	0.1186(19)	0.2301(9)	0.012	1.00000

SI12	0.6887(23)	0.1266(19)	0.7671(9)	0.012	1.00000
O1	0.2482(51)	0.5285(61)	0.1947(28)	0.024	1.00000
O2	0.1632(61)	0.1948(61)	0.0840(23)	0.024	1.00000
O3	0.3895(66)	0.3529(61)	0.1601(28)	0.024	1.00000
O4	0.0218(47)	0.8611(61)	0.0657(23)	0.024	1.00000
O5	0.5132(47)	0.5518(56)	0.2475(28)	0.024	1.00000
O6	0.3791(80)	0.2893(70)	0.4200(24)	0.024	1.00000
O7	0.0769(56)	0.2023(66)	0.1638(28)	0.024	1.00000
O8	0.0338(47)	0.3618(66)	0.0692(23)	0.024	1.00000
O9	0.0991(66)	0.3569(66)	0.1794(28)	0.024	1.00000
O10	0.1913(51)	0.7184(51)	0.0447(28)	0.024	1.00000
O11	0.2504(75)	0.7460(42)	0.2528(23)	0.024	1.00000
O12	0.2215(75)	0.1952(51)	0.2991(23)	0.024	1.00000
O13	0.0462(61)	0.6674(51)	0.1435(19)	0.024	1.00000
O14	0.3844(56)	0.3858(66)	0.2925(23)	0.024	1.00000
O15	0.1300(51)	0.5406(61)	0.0133(37)	0.024	1.00000
O16	0.1305(51)	1.0326(61)	0.0040(37)	0.024	1.00000
O17	0.2241(75)	0.0030(47)	0.2057(23)	0.024	1.00000
O18	0.4772(80)	0.0767(51)	0.6096(28)	0.024	1.00000
O19	0.1954(51)	0.4072(66)	0.3613(23)	0.024	1.00000
O20	0.0838(70)	0.5875(51)	0.2764(28)	0.024	1.00000
O21	0.5816(51)	0.0922(61)	0.6950(19)	0.024	1.00000
C1	0.6775(47)	0.8348(33)	0.5264(33)	0.051	1.33333
C3	0.5805(33)	0.2838(47)	0.5059(42)	0.051	1.33333
C5	0.5795(28)	0.9498(42)	0.5064(37)	0.051	1.33333
C2	0.624(19)	0.601(5)	0.4718(33)	0.051	0.66667
N2	0.529(14)	0.656(14)	0.4117(18)	0.051	0.66667

Note: Realistic s.u. were obtained by multiplying the calculated s.u. with a factor of 4.68 (as provided by the FullProf program after the final stage of the Rietveld refinement). The occupancy factors of C and N atoms are increased to account for the hydrogen atoms bonded to C and N.

S4.5. cif-files

Please see attached files.

1. Structure of RUB-5 polymorph II

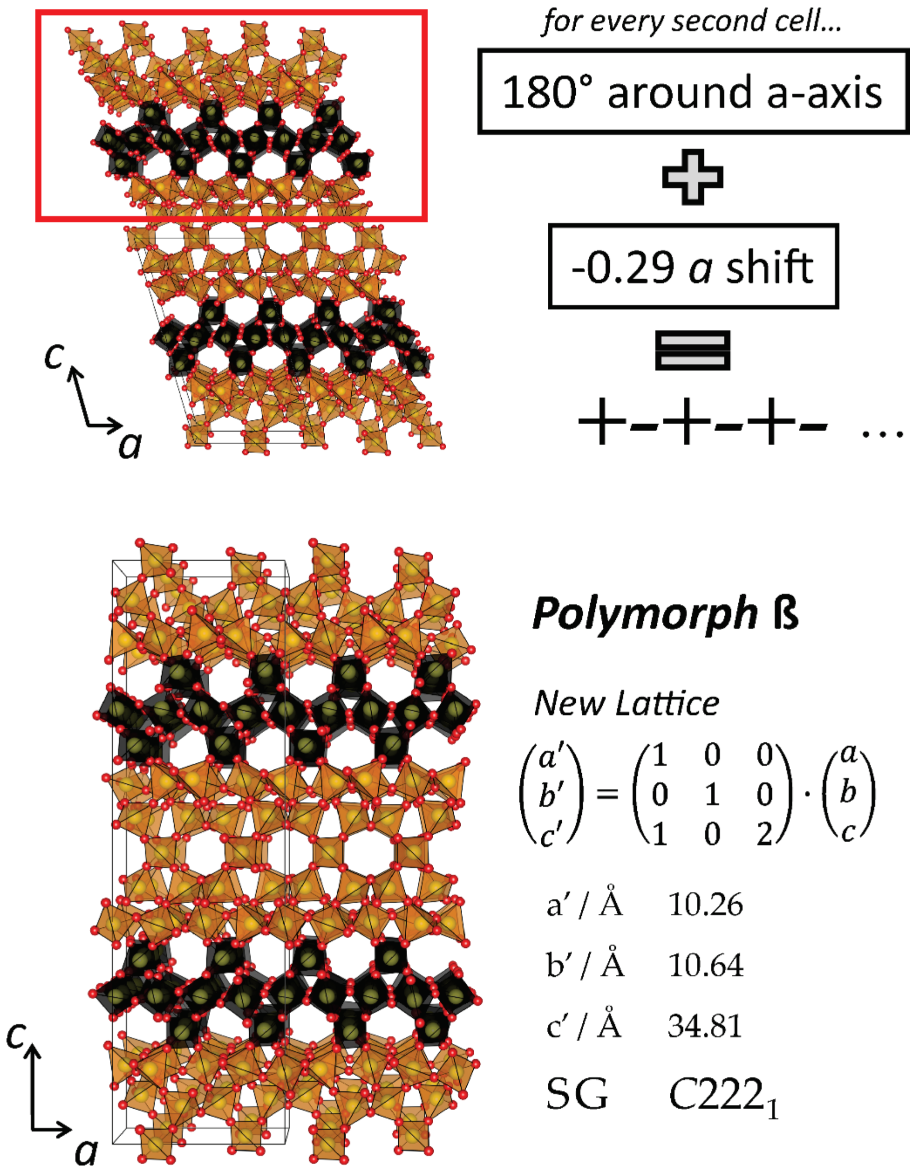

Figure S8 Construction plan to build polymorph II from polymorph I of RUB-5.

2. Disorder: Amount of RUB-6 in RUB-5

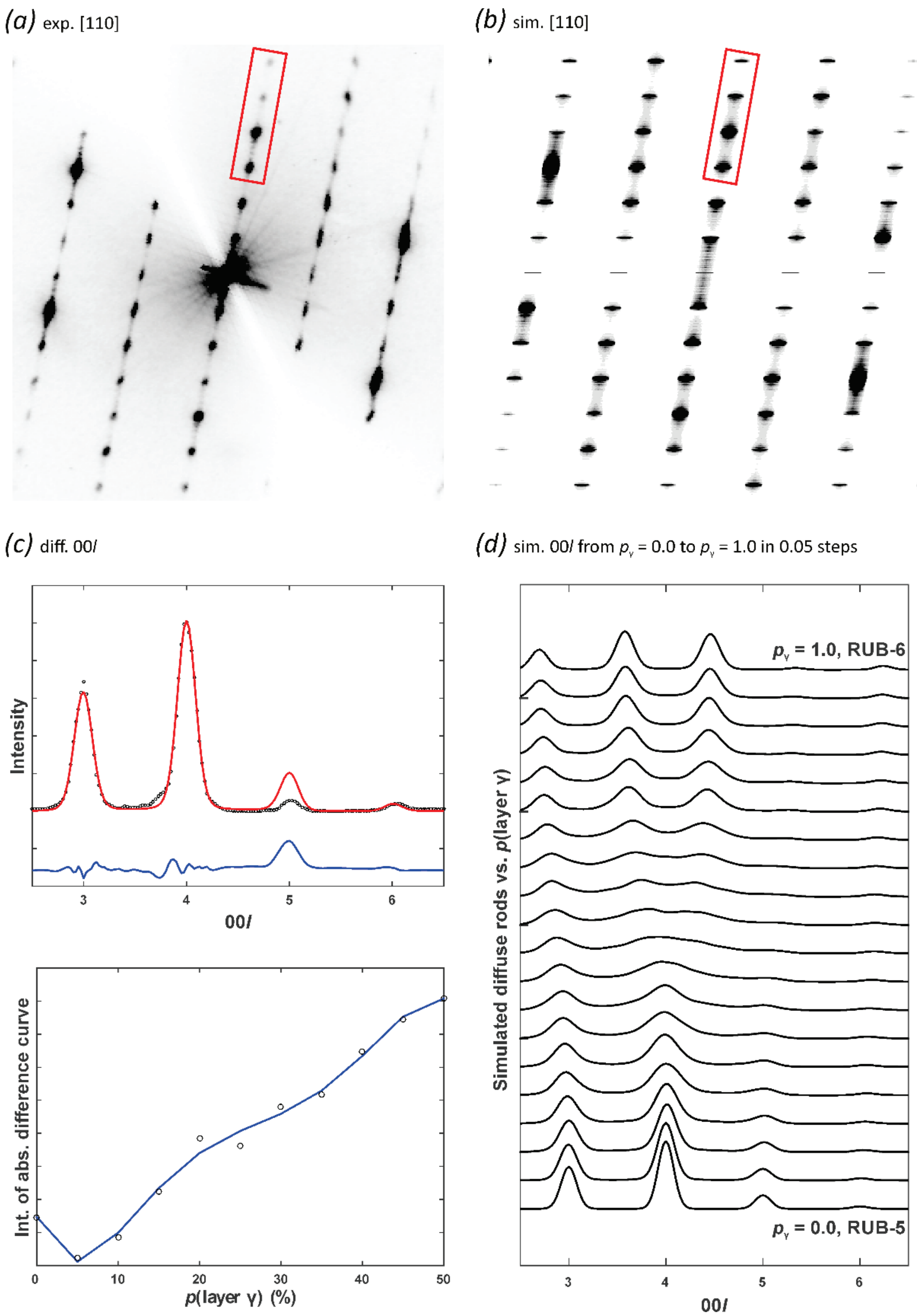

Figure S9 Experimental (a) and simulated (b) diffraction patterns of zone [110] including 00*l* reflections computed by *diffuse_extractor*. Plot of experimental line profile 00*l* (black circles) and the simulated line profile 00*l* (red) of the stacking probability $p_\gamma = 0.05$, the corresponding difference curve (blue) and plot of integrated absolute difference between simulated and experimental line profile taken along 00*l* against stacking probability of layer γ (c). Intensity profiles taken along

corresponding diffraction lines marked in (a) and (b) by red rectangles. The simulated line profiles are plotted for $p_y = 0.00$ to $p_y = 1.00$ in 0.05 steps from bottom to top (d).

3. Comparison of RUB-5 and RUB-6's diffuse scattering

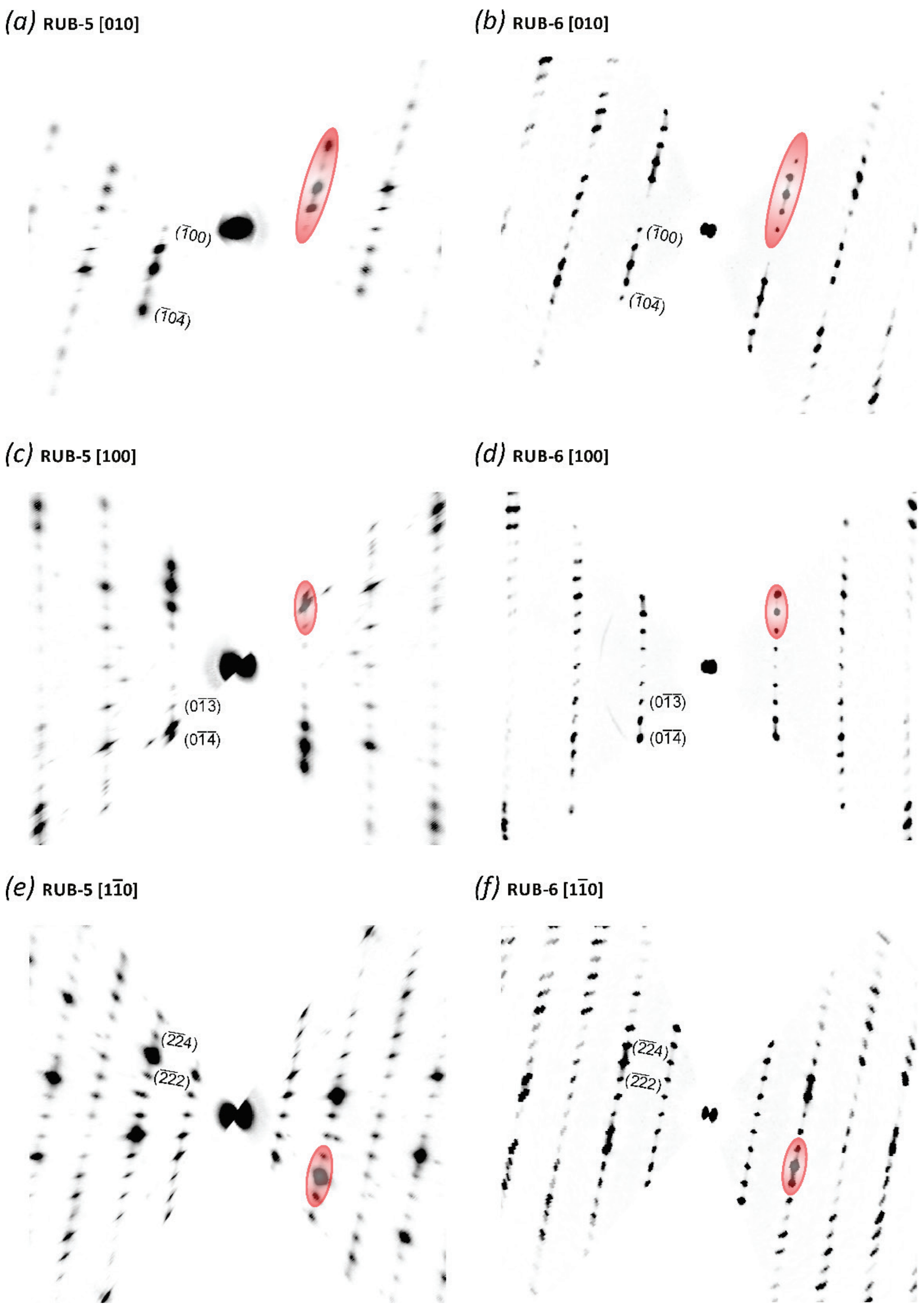

Figure S10 Experimental zones of RUB-5 and RUB-6 ADT datasets, extracted with *diffuse_extractor*, showing comparable diffuse streaks in zones [010] (a, b), [100] (c, d) and [1-10] (e, f). Selected regions are marked in red to indicate the similarity of the two compounds in reciprocal space.
